# Selagibenzophenone B and Its Derivatives: **SelB-1**, a Dual Topoisomerase I/II Inhibitor Identified through In Vitro and In Silico Analyses

**DOI:** 10.1021/acsbiomedchemau.4c00027

**Published:** 2024-07-26

**Authors:** Serhat Dönmez, Ringaile Lapinskaite, Hazal Nazlican Atalay, Esra Tokay, Feray Kockar, Lukas Rycek, Mehmet Özbil, Tugba Boyunegmez Tumer

**Affiliations:** † Graduate Program of Molecular Biology and Genetics, School of Graduate Studies, Canakkale Onsekiz Mart University, Canakkale 17020, Turkey; ‡ Department of Organic Chemistry, Center for Physical Sciences and Technology (FTMC), Akademijos g. 7, Vilnius LT-08412, Lithuania; § Department of Organic Chemistry, Faculty of Science, Charles University, Hlavova 8, 128 43 Praha 2, Czechia; ∥ Department of Molecular Biology and Genetics, Faculty of Sciences and Arts, Balikesir University, Balikesir 10145, Turkey; ⊥ Institute of Biotechnology, Gebze Technical University, Kocaeli 41400, Turkey; # Department of Molecular Biology and Genetics, Faculty of Science, Canakkale Onsekiz Mart University, Canakkale 17020, Turkey

**Keywords:** cancer, dual inhibition, in silico molecular docking, in vitro, topoisomerase

## Abstract

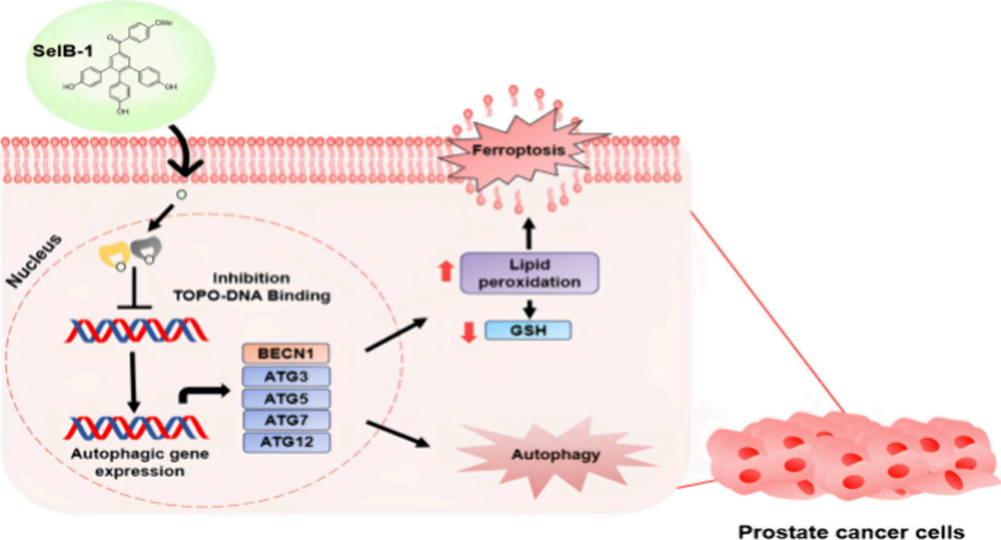

The development of multitargeted drugs represents an innovative approach to cancer treatment, aiming to enhance drug effectiveness while minimizing side effects. Herein, we sought to elucidate the inhibitory effect of selagibenzophenone B derivatives on the survival of cancer cells and dual topoisomerase I/II enzyme activity. Results demonstrated that among the compounds, **SelB-1** selectively inhibited the proliferation and migration of prostate cancer cells while exhibiting minimal effects on healthy cells. Furthermore, **SelB-1** showed a dual inhibitory effect on topoisomerases. Computational analyses mirrored the results from enzyme inhibition assays, demonstrating the compound’s strong binding affinity to the catalytic sites of the topoisomerases. To our surprise, **SelB-1** did not induce apoptosis in prostate cancer cells; instead, it induced autophagic gene expression and lipid peroxidation while reducing GSH levels, which might be associated with ferroptotic death mechanisms. To summarize, the findings suggest that **SelB-1** possesses the potential to serve as a dual topoisomerase inhibitor and can be further developed as a promising candidate for prostate cancer treatment.

## Introduction

Natural product-based compounds are still the main source of marketed anticancer drugs. According to recent records, 247 drugs have been approved for cancer treatment by world authorities (FDA, EMA, etc.) from 1981 to 2019, and more than 50% of approved anticancer drugs are natural product derivatives or biosimilars.^1^ Recognizing the clinical potential inherent in these compounds, it becomes imperative to establish robust synthetic methodologies facilitating not only the synthesis of natural products but also their biomimetics. In many instances, the synthesis of derivatives is the sole means of accessing these compounds.

Plants belonging to the genus *Selaginella* (*Selaginellaceae*) are considered living fossils, boasting an estimated age of 400 million years. Within this genus, over 100 structurally diverse polyphenolic compounds have been identified, encompassing unique entities such as selaginellins and selaginpulvilins.^2^ These compounds contribute to the rich pharmacological profile associated with *Selaginella*, further emphasizing the importance of advancing synthetic strategies to unlock the therapeutic potential of both natural products and their distinctive derivatives.

Selagibenzophenone A (**SelA**), an arylated benzophenone recently isolated by Liu et al., demonstrated inhibitory activity against PDE4D2 with an IC_50_ of 1.04 μM in a cell-free enzymatic assay.^3^ Recently, we reported a modular synthesis of **SelA** and its derivatives, which were further evaluated for various biological effects.^2^ These include inhibition of intracellular PDE4D2, cytotoxic activities toward various cancer cell lines, an inverse agonistic activity on the nuclear receptor RORγ, and an antimicrobial activity. Interestingly, neither **SelA** nor its derivatives revealed a discernible impact on cellular cAMP levels, a surrogate measure of PDE4 activity, in HEK293 cells. Derivatives **SelA-1** and **SelA-2** showed moderate cytotoxicities toward the investigated cancer cell lines (EC_50_ range between 22.0 and 54.7 μM), and for **SelA-2**, the selectivity index increased to 4.1 for the PC3 cell line. The best results were obtained with derivatives **SelA-3** and **SelA-4**. The former showed a promising potency (EC_50_ = 17.7 μM) against HT-29 with a good selectivity index of 8.2, and the latter showed good potency (7.8 μM) against the PC3 cell line with a selectivity index of 3.5.^2^ In 2020, and Tan et al. reported the isolation of a compound with a structural formula corresponding to selagibenzophenone B (**SelB**).^4^ However, we have demonstrated that authors elucidated its structure incorrectly, and in fact, the isolated compound was **SelA**.^5^ Therefore, the biological effect previously associated with **SelB** must be ascribed to **SelA**.^4,6^

**Figure 1:**
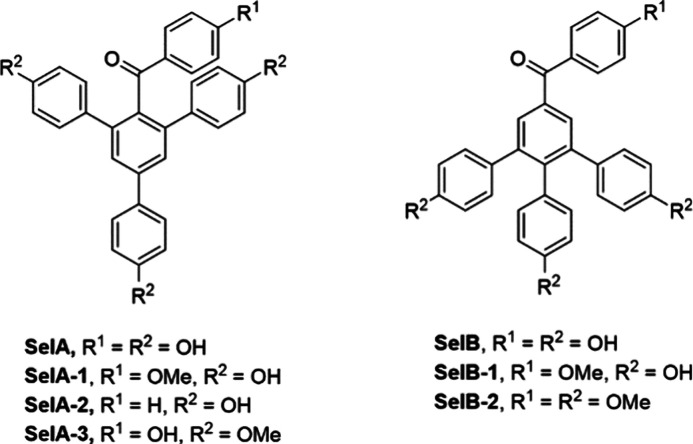
Compounds related to this study. **SelA** and its derivatives **SelA-1–4** and synthetic **SelB** and derivatives **SelB-1** and **SelB-2**. **SelB** was previously mistakenly considered as a natural product, but we proved that its structure was incorrectly elucidated.

In this current report, we present an evaluation of synthetic **SelB** and two derivatives thereof (**SelB-1** and **SelB-2**), which were prepared according to a previous protocol developed by us.^5^ Subsequent to synthesis, these compounds underwent screening to assess their cytotoxic properties and selectivity across diverse cancer cell lines. Notably, **SelB-1** exhibited remarkable potency, with an IC_50_ below 10 μM, and demonstrated a notably high selectivity index against prostate cancer. Further, investigation into the mechanistic underpinnings of its action was conducted. **SelB**, while displaying moderate toxicity in colon and prostate cancer cell lines, holds promising pharmacophores for the derivatization of new potent anticancer agents due to its minimal toxicity level in healthy cell lines.

The compounds featured in this study, identified as having substantial cytotoxic potential, and compounds identified in our prior reports were additionally evaluated for their ability to inhibit topoisomerase enzymes. **SelA** and **SelB** derivatives exhibit a structural similarity to polycyclic groups found in well-known topoisomerase inhibitors like topotecan (**TPT**) and etoposide (**ETP**). Topoisomerases are evolutionarily conserved nuclear enzymes, which play vital roles in DNA replication, transcription, recombination, and chromatin remodeling.^7^ During these processes, DNA strands unwind, leading to the formation of the supercoiled, knotted DNA structures, and these DNA structures are relaxed by short tandemly one-strand (TOPO I) or two-strand breaks (TOPO II). The proactive role of topoisomerases in these fundamental processes is an attractive target for cancer treatment. Consequently, researchers have developed selective inhibitors for both TOPO I and TOPO II, which have been employed in treating various cancers. For instance, FDA-approved TOPO I inhibitors camptothecin and topotecan decreased the colon, lung, prostate, and melanoma cancer cell development both in in vitro and in clinical trials.^8−11^ Additionally, TOPO II inhibitors such as etoposide and doxorubicin, also approved by the FDA, are currently used for lung, breast, ovarian, and prostate cancer treatment.^12−15^ Although selective topoisomerase inhibitors are effective in decreasing prostate cancer cell progression, the compounds have several side effects including hematological toxicity, diarrhea, and neutropenia.^16^ Moreover, inhibiting one type of topoisomerase might inadvertently enhance the activity of the other, leading to drug resistance.^17,18^ Regarding this, dual inhibition of both TOPO I and TOPO II has emerged as a promising strategy to counter the simultaneous increase in topoisomerase activity and to prevent drug resistance. Promising examples of such dual topoisomerase inhibitors include elomotecan, aclurubicin, and TAS-103, both of which are currently undergoing clinical trials (NCT01435096, NCT03045627, NCT04254640, and NCT03181815).^19,20^ To ascertain the potential of compounds, which we have synthesized by using biomimetic approaches, as topoisomerase inhibitors, the inhibitory effects of the compounds on both TOPO I and TOPO II were analyzed. This comprehensive exploration aims to delineate the mechanistic attributes and therapeutic potential of the synthesized compounds in relation to their impact on cellular topoisomerase functions.

## Results and Discussion

### **SelB** Derivatives Selectively Inhibited the Survival of Prostate Cancer Cell Lines

**Figure 2:**
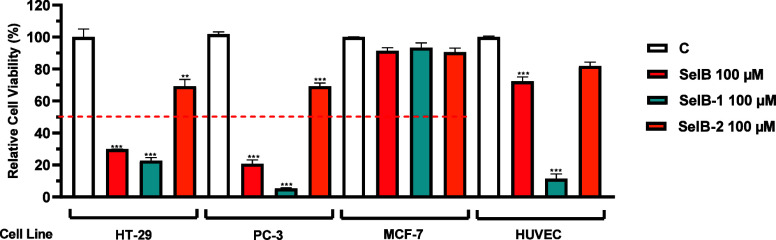
Screening of A 100 μM dose of **SelB** derivatives on HT-29, PC-3, MCF-7, and HUVEC cell lines. C: Control (only DMSO (0.1%)). Each data point is represented as the mean ± SEM obtained from three independent experiments. SEM: Standard error of means. **p < 0.005, ***p < 0.001.

The synthesized compounds were tested for their capability to inhibit cell proliferation in various human cancer cell lines, including MCF-7 (breast carcinoma), PC-3 (prostate carcinoma), and HT-29 (colon carcinoma), as well as in a healthy cell line, HUVEC (human umbilical vein endothelial cells), using the sulforhodamine B (SRB) assay. Initially, a 100 μM dose of the compounds was screened on all cell lines. The results indicated that, apart from **SelB-2**, all compounds significantly reduced the viability of HT-29 and PC-3 cells to below 50%. Importantly, none of the compounds displayed cytotoxic effects on the MCF-7 cell line. Notably, **SelB-1** exhibited a remarkable inhibitory effect, suppressing the proliferation of HT-29, PC-3, and HUVEC cells by 78, 95, and 57%, respectively. Similarly, **SelB** reduced the viability of colon and prostate cancer cells by more than 50% (71 and 80%, respectively) while having no significant effect on healthy cells (28%) (Figure 2).

Hence, **SelB-1** and **SelB** were chosen to determine their half-maximal inhibitory concentration (IC_50_) values on both cancerous and noncancerous cells. Additionally, the selectivity index (SI) of these compounds was calculated by dividing the IC_50_ value obtained for a noncancerous cell line by that for a specific cancer cell line. As shown in Table 1, the compounds selectively inhibited cell proliferation of the prostate cancer cells. The IC_50_ value of the **SelB-1** was found lower than 10 μM on the prostate cancer cells (5.9 μM), and the SI of the compound was 12.3. Furthermore, **SelB-1** showed a good level of cytotoxicity and selectivity against HT-29 cells (IC_50_ value of 17.6 μM, SI: 4.6). On the other hand, **SelB** had the highest SI index for both prostate and colon cancer cells (18.9 and 12.6, respectively). However, the IC_50_ values of the compound on the cells were higher than that of **SelB-1** (Figure S1 and Table 1). The main difference between the structure of the compounds was their number of hydroxyl (−OH) and methoxy (−OMe) groups. In most cases, the addition of the methoxy groups to the structure of the compounds increased the cytotoxic effect.^21^ Consequently, **SelB-1** exhibited a stronger cytotoxic effect than **SelB**, while **SelB-2** displayed the lowest cytotoxic effect despite having the highest number of methoxy groups compared to the others. This may be explained by the possible decrease in the cytotoxic potential of compounds after the replacement of the hydroxyl group with the methoxyl group.^2^

**Table 1:** IC_50_ Values and Selectivity Index (SI) of **SelB-1** and **SelB**

	IC_50_ ± SE (μM)				selectivity index (SI)^a^		
compound	HT-29	MCF-7	PC-3	HUVEC	HT-29	MCF-7	PC-3
**SelB-1**	17.6 ± 3.0	129.7 ± 40.8	5.9 ± 1.0	73.8 ± 18.6	4.19	0.5	12.3
**SelB**	53.4 ± 29.5	1250 ± 392.5	79.9 ± 28.9	1012 ± 471.3	18.96	0.8	12.6

a SI values for each compound have been determined by dividing the IC_50_ values of the healthy cell line (HUVEC) by the IC_50_ value of the cancerous cell lines (HT-29, MCF-7, and PC-3).

**Figure 3:**
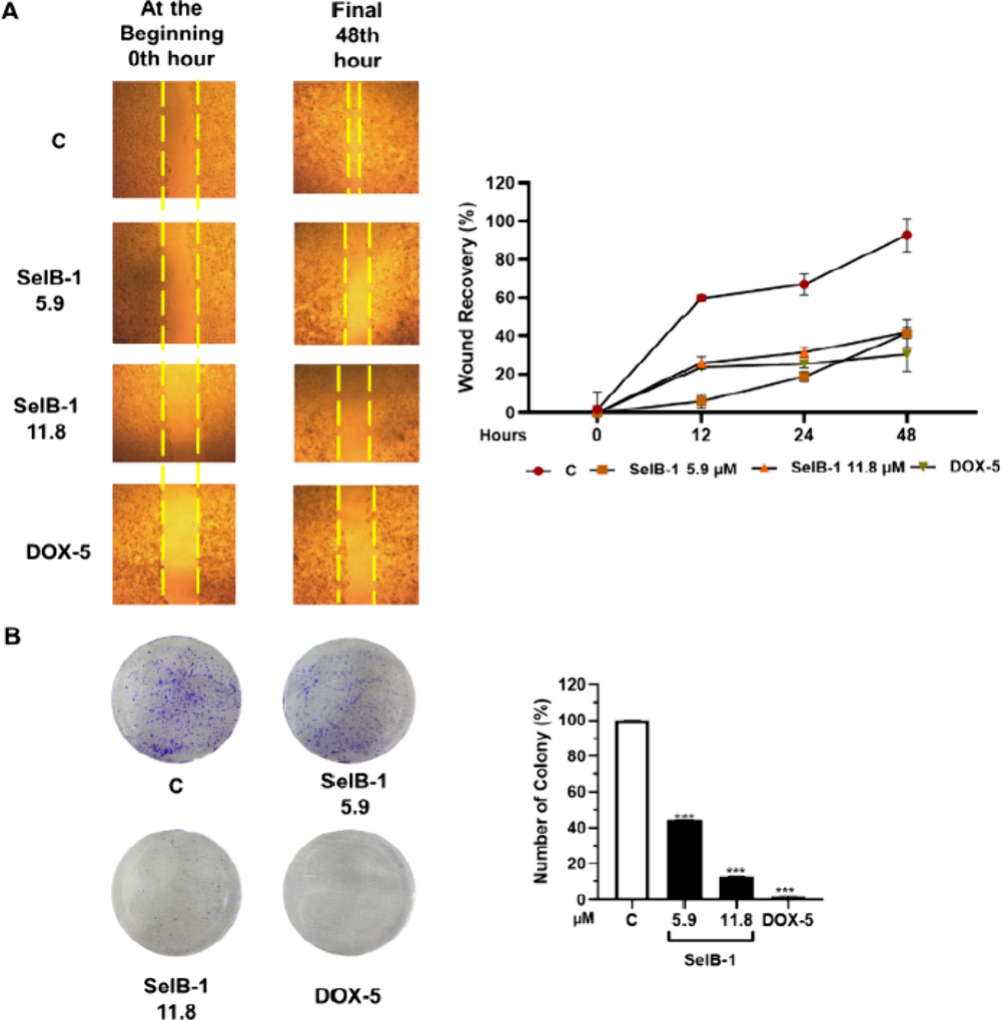
Effect of **SelB-1** on wound recovery (A) and colony formation (B) of PC-3 cells. C: Control (only DMSO (0.1%)). DOX-5: Doxorubicin 5 μM (positive control as a chemotherapeutic drug). Each data point is represented as the mean ± SEM obtained from three independent experiments. SEM: Standard error of means. *p < 0.02, **p < 0.005, and ***p < 0.001.

According to the in vitro cell cytotoxicity assay, **SelB-1** was selected as a lead compound due to its promising potency and high selectivity against prostate and colon cancer cell lines. Subsequently, the impact of the compound on the proliferation and migration capabilities of prostate and colon cancer cells was assessed through colony formation and wound healing assays. Notably, IC_50_ and two-time IC_50_ doses of **SelB-1** significantly reduced the wound recovery of PC-3 cells by 58 and 59%, respectively, at the end of 48 h (Figure 3A). Similarly, the same doses of **SelB-1** application significantly inhibited colony formation of PC-3 cells (Figure 3B). Additionally, IC_50_ and two times IC_50_ doses of **SelB-1** significantly reduced the wound recovery of HT-29 cells by 10 and 56%, respectively. (Figure S2). These results suggest that **SelB-1** application may effectively alter the invasiveness of prostate and colon cancer cells.

### Topoisomerase I and II Enzyme Inhibition Assays

Recently, it has been implicated that the compounds synthesized from *Selaginella* plant species effectively hinder cancer cell growth with a minimal cytotoxic effect on healthy cell lines.^2,22,23^ Notably, most of the isolated components are highly proficient in inhibiting phosphodiesterase 4D (PDE4D) isoforms, which break down cytosolic cyclic adenosine monophosphate (cAMP).^3,24−26^ Furthermore, the expression levels of the enzymes were shown to be increased in prostate cancer patients; therefore, it is proposed to be used as a biomarker for prostate cancer progression.^27^ Similar to the previously isolated compounds, the **SelA** derivatives **SelA-2**, **SelA-3**, and **SelA-4**, which we synthesized previously, demonstrated significant inhibition of cancer cell viability. Furthermore, the compounds showed the most promising effect on the prostate cancer cell line (with respective EC_50_ values of 43.4–7.8 μM). To our surprise, even at a concentration of 10 μM, the compounds were not able to show significant inhibitory effects on the recombinant PDE4 enzyme activity. Accordingly, the potential targets of these synthesized compounds have remained undisclosed.

**Figure 4:**
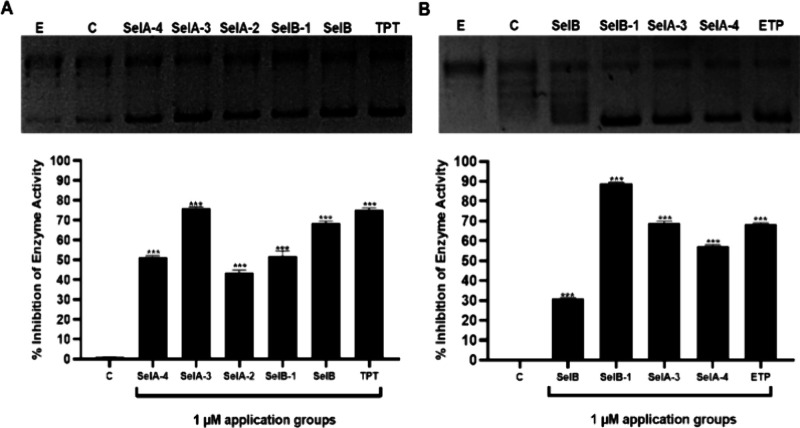
**SelA** and **SelB** derivatives inhibited dual TOPO I (A) and TOPO II (B). Lane E: pHOT1 plasmid 250 ng + topoisomerase enzyme (2U for topoisomerase I (left), 10U for topoisomerase II (right)). Lane C: pHOT1 plasmid 250 ng + topoisomerase enzyme (2U for topoisomerase I (left), 10U for topoisomerase II (right)) + DMSO (1%). Other lanes: pHOT1 plasmid 250 ng + topoisomerase enzyme (2U for topoisomerase I (left), 10U for topoisomerase II (right)) + 1 μM of compounds. Each data point is represented as the mean ± SEM obtained from three independent experiments. SEM: Standard error of means. *p < 0.02, **p < 0.005, and ***p < 0.001.

Targeting multiple biological molecules with one compound is a recently used approach for cancer treatment, for instance, dual topoisomerase I (TOPO I) and II (TOPO II) inhibition. Topoisomerase enzymes play a crucial role in DNA replication and transcription, and their overactivation has been linked to the proliferation of cancer cells.^28^ Consequently, various agents have been developed to inhibit topoisomerases, many of which contain multiple benzene rings.^29,30^**SelA** (**SelA-2**, **SelA-3**, and **SelA-4**) and **SelB** (**SelB** and **SelB-1**) derivatives contain polycyclic groups similar to the topoisomerase inhibitors such as topotecan (**TPT**) and etoposide (**ETP**). Considering this similarity, we hypothesized that these compounds might target topoisomerase enzymes. In order to validate this hypothesis, the TOPO I and TOPO II inhibitory effects of the compounds were analyzed by using TOPOGEN topoisomerase I (TOPO I) and II (TOPO II) enzyme assay kits. As shown in Figure 4A, **SelA-3** showed the highest inhibitory activity (∼76%), surpassing the well-known TOPO I inhibitor **TPT** (75%). Moreover, **SelA-4**, **SelB-1**, and **SelB** inhibited TOPO I enzyme activity higher than 50% (51, 52, and 68%, respectively). The compounds with inhibitory effects on TOPO I were also analyzed for TOPO II. Accordingly, **SelA-3** and **SelB-1** inhibited TOPO II enzyme activity more effectively than the established TOPO II inhibitor **ETP** (89, 69, and 68%, respectively). On the other hand, **SelA-4** showed a 58% inhibitory effect on TOPO II enzyme activity (Figure 4B). In summary, **SelB-1**, **SelA-4**, and **SelA-3** appear to have the potential to act as a dual inhibitor of both TOPO I and II, whereas **SelB** might be a selective TOPO I inhibitor.

### Molecular Docking and Molecular Dynamics (MD) Simulations

Following the enzyme inhibition assay, the interactions between the most potent compounds (with inhibitory activity exceeding 50%) and topoisomerases were examined using computational methods. Initially, the crystal structures of TOPO I (PDB ID 1sc7)^31^ and TOPO II (PDB ID 4j3n)^32^ were subjected to classical molecular dynamics (MD) simulations utilizing GROMACS 5.1.4 software. The crystal structures contain DNA duplexes, and the simulations were performed by the protein–DNA complex. The simulations were conducted until the backbone root-mean-square deviation (RMSD) values of each structure reached equilibrium. TOPO I and TOPO II were subjected to 40 and 30 ns (ns) production simulations, respectively (Figure S3). The most representative protein structures representing the equilibrated protein structures were taken from the last 10 ns of the simulations.

After protein and ligand preparation, molecular docking analyses were conducted using YASARA structure software (generally use YASARA for molecular graphics and modeling with the old OpenGL graphics engine),^33^ which utilizes AutoDock Vina^34^ for molecular docking simulations. All the results obtained from molecular docking analyses are summarized in Table 2. Notably, all compounds were bound to the TOPO I with higher affinity than the reference compound **TPT**. Among these compounds, **SelB** showed the strongest binding to the two different regions of the TOPO I (−10.2/-10.1 kcal/mol). Additionally, **SelB-1** and **SelA-3** bound the protein with similar binding affinities compared to **SelB** (−9.9 and −9.8 kcal/mol, respectively). Similarly, in the case of TOPO II, **SelB-1** and **SelA-3** yielded the same binding affinities to the TOPO II (−9.1 kcal/mol). However, **ETP** bounded to the protein stronger than the compounds (−10.23 kcal/mol). Conversely, **SelA-4** yielded the smallest binding affinity to TOPO I and II compared to other compounds (−9.1 and −8.6 kcal/mol, respectively) (Table 2).

**Table 2:** Binding affinities (left) and binding free energies (right) of SelA and SelB derivatives to TOPO I and II

	molecular docking		molecular dynamics simulations	
	binding affinity (kcal/mol)		binding free energy (kcal/mol)	
compound	TOPO I	TOPO II	TOPO I	TOPO II
**SelB**	–10.2/–10.1		–10.8 ± 0.2/–13.6 ± 0.3	
**SelB-1**	–9.9	–9.2	–13.6 ± 0.2	–13.2 ± 0.2
**SelA-3**	–9.8	–9.1	–13.5 ± 0.3	–14.5 ± 0.5
**SelA-4**	–9.1	–8.6	–12.5 ± 0.3	–14.9 ± 0.2
**TPT**	–8.9		–8.9 ± 0.2	
**ETP**		–10.2		–12.7 ± 0.5

Following the molecular docking analyses, the best docking poses were subjected to MD simulations to assess the stability of the compounds on the binding sites. Fifty ns long simulations were performed for all protein–ligand complexes, and RMSD values of the proteins in the complexes indicated that equilibrium was reached (Figures S4 and S5). Furthermore, lRMSD values for each ligand were determined around 0.1 nm or lower, which indicated that all the ligands stayed mostly in their binding sites during simulations (Figures S6 and S7). In terms of binding affinity, all the compounds remained in the binding sites stronger than the reference compounds (Table 2). Surprisingly, both poses of the **SelB** remained in the binding sites of TOPO I with relatively high binding energies (−10.8 and −13.6 kcal/mol) (Table 2). This suggests that **SelB** might be capable of binding to two different regions of TOPO I.

**Figure 5:**
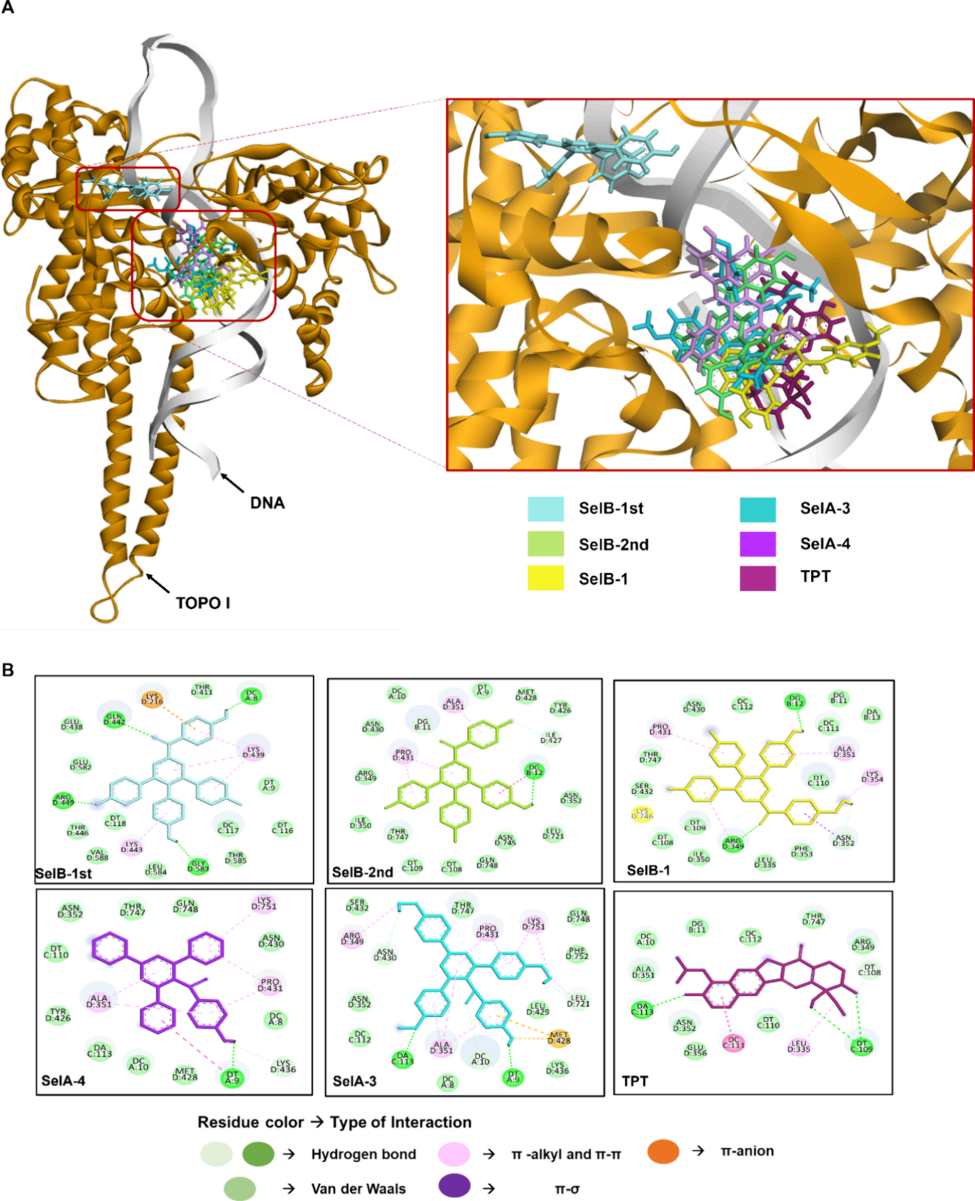
Binding mode (A) and interactions (B) between **SelB**, **SelB-1**, **SelA-4**, **SelA-3**, **TPT**, and TOPO I at the end of MD simulations.

**Figure 6:**
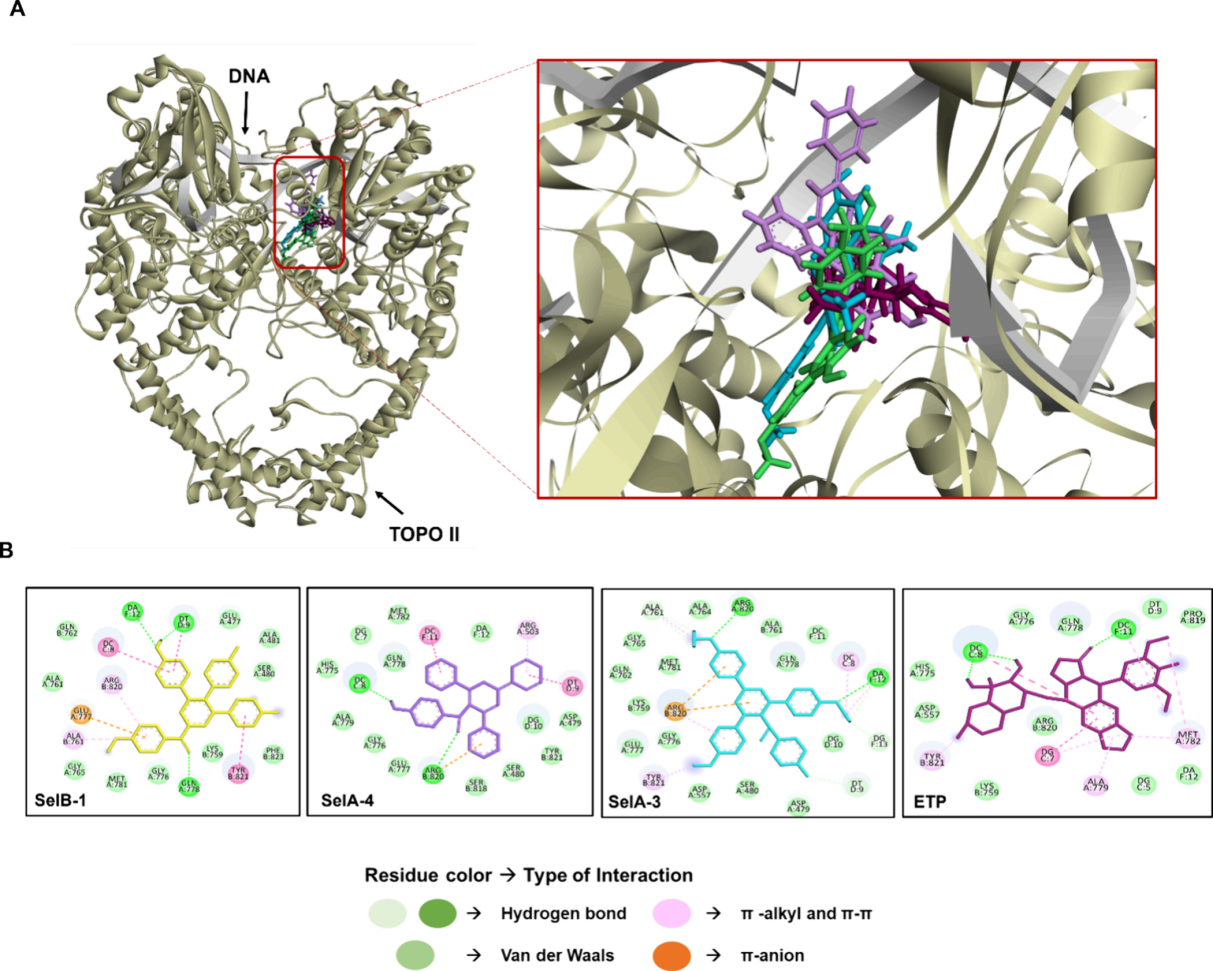
Binding mode (A) and interactions (B) between **SelA-3**, **SelA-4**, **SelB-1**, **ETP**, and TOPO II at the end of MD simulations (B).

In addition to the binding affinity and binding free energy calculations, the interactions between the compounds and topoisomerases were visualized by Discovery Studio Client (Figures 5 and 6 and Figures S8 and S11). Specifically, in both molecular docking and MD simulations, **SelB-1** and the second-best pose of **SelB** interacted with Arg 349, Ala 351, Lys 354, and Pro 431 residues of TOPO I (Figure 5A,B and Figure S7). Similarly, **SelA** derivatives, **SelA-3** and **SelA-4**, interacted with Dc 8, Dt 9, Dc 10, Da 113, Arg 349, Ala 351, Met 428, Pro 431, and Lys 751 residues of TOPO I through hydrogen bonding or hydrophobic interactions (Figure 5A,B and Figure S8). On the other hand, the best docking pose of **SelB** yielded interactions with Dc 8, Lys 216, Lys 439, and Arg 449 residues of TOPO I (Figure 5A,B and Figure S8). The **SelA** and **SelB** derivatives (apart from the first pose of **SelB**) interacted with similar residues, and the compounds retained their hydrogen bonding and hydrophobic interactions during the simulations (Figure S9). Additionally, the binding of the **SelA** and **SelB** derivatives slightly decreased the root-mean-square fluctuation (RMSF) values of their respective binding residues compared to ligand-free TOPO I (Figure S10). However, the change in the position of two benzene rings between **SelA** and **SelB** derivatives (Figure 1) led to variations in the types of interactions. For instance, both **SelA-3** and **SelA-4** yielded a higher number of π–π and π–alkyl interactions but a smaller number of van der Waals interactions with TOPO I compared to the **SelB** and **SelB-1** at the end of MD simulations (Figure 5A). Moreover, the change in the type of interaction also resulted in decreased stability of the **SelA** derivatives into the binding site of TOPO I compared to **SelB** derivatives in terms of binding free energy (Table 2). Notably, **SelA-3** stayed in the binding site of the TOPO I with similar energy to the second pose of **SelB** and **SelB-1**. This similarity could be attributed to the **SelA-3** forming the highest number of hydrogen bonding (5) and π–π and π–alkyl interactions (8) while showing the smallest number of van der Waals interactions (9) compared to all derivatives (Figure 5 and Figure S7). **SelB-1** and the second pose of **SelB** showed the same number of hydrogen bonding (3), van der Waals (14), and π–π and π–alkyl interactions (4), and both remained in the TOPO I binding site with the same energy (Table 2, Figure 5, and Figure S8).

The best pose of **SelB** showed a higher number of hydrogen bonding interactions (5) but smaller van der Waals interactions (9) compared to the second-best pose, which could explain the 2.8 kcal/mol lower binding energy of the best pose of **SelB** (Table 2 and Figure 5B).

All the compounds interacted with the DNA binding site of TOPO II, specifically with residues Gly 76, Glu 777, Tyr 821, and Arg 820 and DNA bases T 9 and A 12, both in molecular docking and MD simulations (Figure 6 and Figure S11). Furthermore, the compounds retained their interactions during the MD simulations (Figure S12). Additionally, binding of the compounds increased RMSF values of the residues comprising binding sites (Figure S13).

In contrast to TOPO I, A derivatives exhibited a stronger binding to TOPO II compared to **SelB-1** in terms of binding free energy. The variation in the positions of the benzene rings likely enabled **SelA-3** and **SelA-4** to be well-positioned in the TOPO II binding site, resulting in a higher number of interactions between **SelA** derivatives and TOPO II compared to **SelB-1** (Figures 1 and 5). Among the **SelA** derivatives, **SelA-3** showed a higher number of hydrogen bonding and hydrophobic interactions with TOPO II compared to **SelA-4**. Conversely, **SelA-4** remained in the binding site of TOPO II with higher energy than **SelA-3** (0.4 kcal/mol) (Table 2). The enhanced stability of **SelA-4** could be attributed to the scoring function of the PRODIGY-LIGAND server. The binding energy calculation between a ligand and a protein is not determined solely by the number of hydrogen bonds, π–π interactions, or van der Waals interactions but also considers atomic contacts between the ligand and protein within a 10.5 Å range.^35,36^ Regarding this, **SelA-4** may have had a higher number of atomic contacts than **SelA-3** within the corresponding range, leading to the highest binding free energy observed (Table 2). On the other hand, **SelB-1** stayed in the binding site of TOPO II with lower energy than **SelA** derivatives (Table 2). Even though the compound showed a higher number of π–π and π–alkyl interactions (5) compared to **SelA** derivatives, the compound only interacted with 10 residues through van der Waals interactions fewer than **SelA-3** and **SelA-4** (which had 14 and 13 van der Waals interactions, respectively) (Figure 6B). Consequently, the difference in the number of van der Waals interactions likely accounted for the 1.3 and 1.7 kcal/mol binding energy difference between **SelB-1** and **SelA** derivatives (Table 2). Altogether, our **SelB-1**, **SelA-3**, and **SelA-4** could be dual TOPO I and II inhibitors, while **SelB** selectively inhibited TOPO I activity. In summary, based on in silico molecular docking and MD simulations, **SelB-1**, **SelA-3**, and **SelA-4** showed interactions with the DNA binding sites of both TOPO I and II. However, the effects of the **SelA** and **SelB** derivatives on binding site of the TOPO I and II were different. While binding of the compounds stabilized the binding site of the TOPO I, the opposite effect was observed on the TOPO II binding site according to the RMSF calculations (Figures S10 and S13). In contrast, **SelB** interacted with two distinct sides of TOPO I.

### **SelB-1** Application Did Not Induce Apoptosis but Increased Autophagic Gene Expression

**Figure 7:**
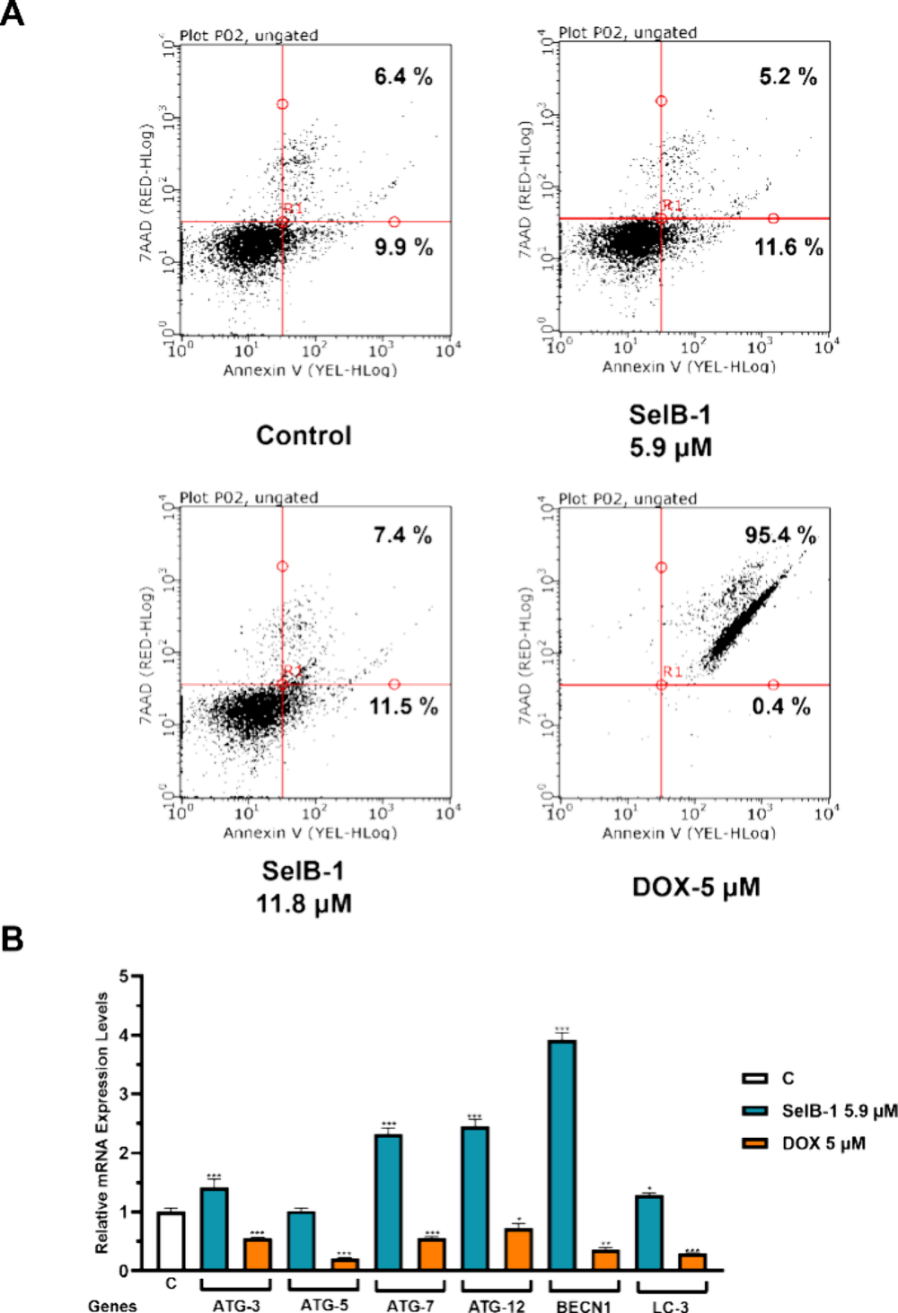
Effect of **SelB-1** on apoptotic and autophagic cell death. Annexin V/PI staining of PC-3 cells after treatment with **SelB-1** for 48 h (A). The bottom left quarter indicates alive cells; the bottom right quarter indicates early apoptotic cells; the upper right quarter indicates late apoptotic cells; the upper left quarter indicates necrotic cells. Effect of **SelB-1** on ATG-3, ATG-5, ATG-7, ATG-12, BECN1, and LC-3 gene expression levels on prostate cancer cells (B). C: Control (only DMSO (0.1%)). DOX-5: Doxorubicin 5 μM (positive control). Each data point is represented as the mean ± SEM obtained from three independent experiments. SEM: Standard error of means. *p < 0.02, **p < 0.005, and ***p < 0.001.

The previous study by Lapinskaite et al. examined the cytotoxic effects of **SelA-3** and **SelA-4** on both cancer and healthy cell lines.^2^ It was found that **SelA-4** inhibited prostate cancer cell viability with an IC_50_ value of 7.8 μM and a selectivity index (SI) of 3.5. Conversely, the same inhibitory effect was not observed for **SelA-3** in prostate cancer cells. Herein, **SelB-1** demonstrated higher cytotoxicity on cancer cells than **SelA-4** in terms of both IC_50_ and SI (Table 1). Consequently, the effect of **SelB-1** on cell death and cell cyle arrest was investigated by flow cytometry analyses. Accordingly, **SelB-1** treatment slightly increased the percentage of apoptotic cells; however, this induction was not significant compared to the control group (Figure 7A). Furthermore, **SelB-1** application did not induce cell cycle arrest on prostate cancer cells (Figure S14). Hence, **SelB-1** might not induce apoptosis and cell cycle arrest in prostate cancer cells.

The enzyme inhibition assays indicated that **SelB-1** has the potential to act as a dual TOPO I and II inhibitor. Previous studies showed that topoisomerase inhibitors could induce apoptotic, necrotic, or autophagic cell death.^37−39^ However, in the flow cytometry analyses, **SelB-1** did not induce an increase in either apoptotic or necrotic cell percentages (Figure 7A). Thus, we investigated the effect of **SelB-1** on autophagic cell death through gene expression analysis. As shown in Figure 7, the **SelB-1** application increased the expression level of ATG-3, ATG-7, ATG-12, and BECN1 while not affecting the ATG-5 and LC-3 expression levels. Specifically, at the IC_50_ dose, the compound increased the expression levels of ATG-3, ATG-7, ATG-12, and BECN1 genes by 1.3-fold, 2.3-fold, 2.4-fold, and 3.9-fold, respectively (Figure 7B). According to the findings, **SelB-1** application might partially induce autophagy in prostate cancer cells. (Table 2). Altogether, our **SelB-1**, **SelA-3**, and **SelA-4** could be dual TOPO I and II inhibitors, while **SelB** selectively inhibited TOPO I activity. In summary, based on in silico molecular docking and MD simulations, **SelB-1**, **SelA-3**, and **SelA-4** showed interactions with the DNA binding sites of both TOPO I and II. In contrast, **SelB** interacted with two distinct sides of TOPO I.

### **SelB-1** Might Induce Ferroptosis in Prostate Cancer Cells

Induction of the autophagy-related genes is not only associated with autophagy but also is linked to ferroptosis.^40,41^ For instance, ATG7, a gene known for its function in autophagy, has been found to be involved in ferroptosis.^42^ ATG7 may regulate ferroptosis by altering cellular breakdown processes. Autophagy, which is regulated by ATG7, has been shown to increase ferroptosis by promoting the destruction of ferritin, a protein that regulates iron storage.^43^ Furthermore, AMPK has been demonstrated to phosphorylate Beclin-1, another essential protein involved in autophagy, in the context of ferroptosis. Beclin-1 can contribute to ferroptosis by binding to and diminishing the action of system xc-, an antiporter that regulates cysteine import.^44^ Based on the findings of the study, it was observed that the **SelB-1** led to 2.3- and 3.9-fold increases in ATG-7 and Beclin-1 levels, respectively. These significant increases imply that the **SelB-1** might have the potential to induce cell death through ferroptosis. Ferroptosis is iron-dependent cell death, which is distinct from apoptosis, autophagy, and necrosis. Alteration of the iron metabolism in the cells leads to a reduction in the glutathione (GSH) levels and induction of lipid peroxidation. Regarding this, GSH depletion and induction of lipid peroxidation are two signs of ferroptosis.^45,46^

**Figure 8:**
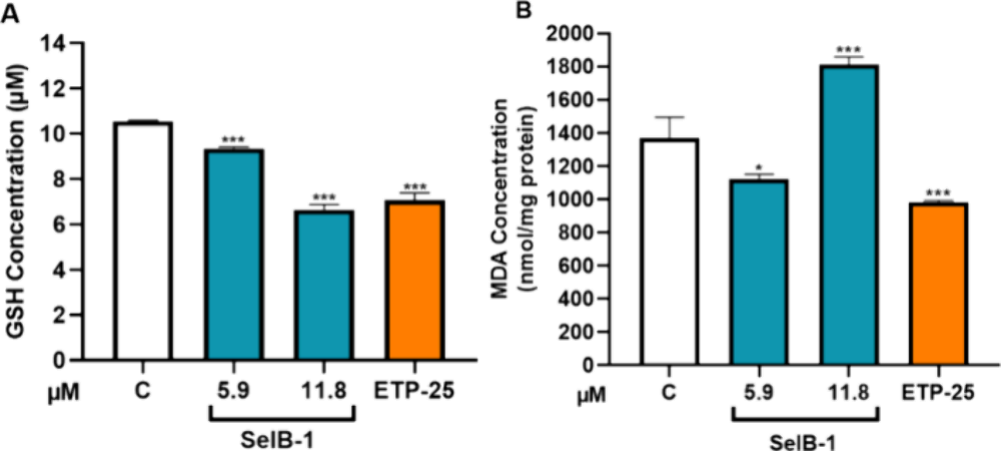
Effect of **SelB-1** on the GSH level (A) and lipid peroxidation (B) in prostate cancer cells. C: Control (only DMSO (0.1%)). ETP-25: Etoposide 25 μM (positive control). Each data point is represented as the mean ± SEM obtained from three independent experiments. SEM: Standard error of means. *p < 0.02, **p < 0.005, and ***p < 0.001.

To explore the potential ferroptotic effect of **SelB-1**, its impact on glutathione (GSH) levels and lipid peroxidation in prostate cancer cells was investigated. **SelB-1** application altered GSH levels in the prostate cancer cells. Accordingly, two times IC_50_ doses of **SelB-1** decreased the GSH level of prostate cancer cells more significantly than etoposide (44 and 37%, respectively) (Figure 8A).

During ferroptosis, polyunsaturated fatty acids undergo peroxidation, ultimately resulting in the formation of malondialdehyde (MDA). Consequently, MDA serves as a marker for lipid peroxidation.^47^ According to the results shown in Figure 8B, two times IC_50_ doses of **SelB-1** induced lipid peroxidation compared to the control group (about 1.2 times).

## Conclusions

In this study, we evaluated the anticancer potency/selectivity and the dual TOPO I/II inhibitory capacity of **SelB** derivatives through a combination of in vitro and computational analyses. Among the tested compounds, **SelB-1** showed potent and selective antiproliferative activity against prostate cancer cells. Furthermore, the compound effectively altered the migration and colony formation capability of the prostate cancer cells. Additionally, even at low doses, **SelB-1** demonstrated a dual inhibitory effect on both topoisomerase I and II enzymes. In addition to **SelB-1**, derivatives of **SelA**, specifically **SelA-3** and **SelA-4**, were also found to be effective in inhibiting both topoisomerases. Through molecular docking and MD simulations, we determined that the compounds could exert their inhibitory effects by binding to the DNA binding sites of both TOPO I and II enzymes. Intriguingly, **SelB-1** did not induce apoptotic cell death on prostate cancer cells. Instead, the compound triggered the expression of autophagic genes, increased lipid peroxidation (LPO), and reduced the level of glutathione (GSH). Consequently, further investigations are required to gain a comprehensive understanding of the compound’s impact on autophagic and ferroptotic cell death pathways. Moreover, **SelB-1** demonstrated a promising antiproliferative effect on colon cancer cells. However, the current study did not provide a detailed discussion about the compound’s impact on colon cancer cell death.

Overall, our findings indicate that selagibenzophenones might serve as innovative and multitargeted pharmacophores for the development of potent antiprostate cancer agents.

## Experimental Section

All cells were obtained from the American Type Culture Collection (ATCC; Manassas, VA, USA). Dulbecco’s modified Eagle medium (DMEM; 4.5 g/L glucose), fetal bovine serum (FBS), penicillin-streptomycin solution, trypsin-EDTA, a Qubit RNA BR assay kit, and a high-capacity cDNA reverse transcription kit were purchased from Gibco/Thermo Fisher Scientific (Waltham, MA, USA). Sulforhodamine B (SRB), l-glutamine, propidium iodide (PI), and 5,5′-dithiobis(2-nitrobenzoic acid) (DTNB) were obtained from Sigma-Aldrich (St. Louis, MO, USA). A total RNA purification plus kit was purchased from Norgen Biotek Corporation (Thorold, ON, Canada). Topoisomerase I and II enzyme inhibition kits were obtained from TopoGEN (Buena Vista, CO, USA). A Guava Nexin reagent (Guava Nexin reagent for flow cytometry kit) was purchased from Luminex Corporation (Austin, USA).

### Cell Lines and Culture Conditions

HT-29 (human colorectal adenocarcinoma cell line), MCF-7 (human breast cancer cell line), PC-3 (human prostate cancer cell line), and HUVEC (human umbilical vein endothelial cells) were cultured and maintained in DMEM containing 10% FBS and 100 U/mL penicillin-streptomycin. All cell lines were incubated at 37 °C under a saturating humidity atmosphere of 5% CO_2_ and 95% air. Cells were passaged by tyripsin-EDTA when they reached to 60–70% confluency.

### Cytotoxicity Assays

The effect of compounds on cell viability was determined by a sulforhodamine B (SRB) assay as described previously.^2^ Briefly, all cells were seeded in 96-well plates at the concentration of 5 × 10^4^ cells/well and incubated for 24 h. Then, various doses of (between 1 and 100 μM) **SelB** derivatives (in dimethyl sulfoxide (DMSO) (0.1%)) were applied to the seeded cells for 48 h. The percentage of viable cells was evaluated by the SRB assay.

### Cell Proliferation and Cell Migration Assays

The effects of **SelB-1** on cell proliferation and cell migration were determined as described previously.^48^ For the cell proliferation assay, PC-3 cells were seeded into 6-well plates at 1000 cells/well density and incubated for 24 h. After cell attachment, cells were treated with determined doses (IC_50_ and 2-fold IC_50_) of **SelB-1** for 48 h. Then, the medium was discarded and replaced every 2–3 days until colony formation (approximately 50 cells/colony) was observed in wells. The number of colonies was determined by ImageJ software.

In the cell migration assay, both cells were seeded into 24-well plates at a 7.5 × 10^5^ cells/well density and incubated for 24 h. At the end of incubation, cells were scraped in the middle of a well from up to the bottom side with a pipet tip to create a wound. Then, cells were treated with determined doses of **SelB-1** (IC_50_ and 2-fold IC_50_). Cell images were taken with Launch ImageFocus 4 software with the integration of a Euromex inverted microscope at 0, 12, 24, and 48 h. The wound closure was analyzed using ImageJ software.

### Topoisomerase I and II Enzyme Inhibition Assays

The topoisomerase I inhibitory properties of the **SelA** and **SelB** derivatives were evaluated by a TopoGEN topoisomerase I inhibition assay kit (TG1015-3A). Briefly, a topoisomerase I enzyme (2 U) was preincubated with or without compounds at 37 °C for 30 min in the reaction buffer (1 mM Tris–HCl pH 7.9, 0.1 mM EDTA, 0.015 M NaCl, 0.1% bovine serum albumin, 0.01 mM spermidine, and 5% glycerol). After incubation, the reaction volume was completed to 20 μL by the addition of 250 ng of a pHOT1 plasmid. The reaction mixture was incubated at 37 °C for 30 min. Then, the reaction was stopped by the addition of 4 μL of a stop solution (0.125% bromophenol blue, 25% glycerol, and 5% sarkosyl).

A TopoGEN topoisomerase II inhibition assay kit (TG1001-3A) was used to detect the effect of the compounds on topoisomerase II enzyme activity. Accordingly, 250 ng of the pHOT1 plasmid and the topoisomerase II enzyme (10 U) were incubated with or without compounds at 37 °C for 30 min in the reaction buffer (0.05 M Tris-Cl, pH 8.0, 0.15 M NaCl, 10 mM MgCl_2_, 2 mM ATP, 0.5 mM dithiothreitol, and 0.45 mM BSA). The reaction was stopped by the addition of 5 μL of stop solution (0.125% bromophenol blue, 25% glycerol, and 5% sarkosyl).

DNA samples were electrophoresed on a 1% agarose gel at 100 V for 1 h with a running buffer of TAE (Tris-acetate-EDTA). The gel was stained with 1 μg/mL SYBR Green (21414) containing the TAE buffer for 30 min. DNA bands were visualized under UV light and analyzed by Image Studio v.5.2.

### In Silico Analyses

#### Molecular Dynamics (MD) Simulations

The protein structures of topoisomerase I (PDB ID 1sc7)^31^ and topoisomerase II (PDB ID 4j3n)^32^ were subjected to classical molecular dynamics simulations to obtain well-equilibrated protein structures. The 3D structure of both topoisomerase I and topoisomerase II includes DNA duplexes, with lengths of 22 and 20 base pairs, respectively. Regarding this, DNA duplexes were retained within their respective structures, and simulations were conducted involving protein–DNA complexes. The simulations were carried out utilizing GROMACS 5.1.4 software^49^ and the AMBER03 force field.^50^ Briefly, topoisomerase I and II proteins were placed in a cubic box with dimensions of 15 × 15 × 15 Å and 17 × 17 × 17 Å, respectively. Proteins were solvated with water molecules treated with a single point charge (SPC) model,^51^ and sodium chloride ions were placed into the boxes to neutralize the charge of the system. Then, the system was subsequently energy-minimized. After that, 100 ps MD production simulations were carried out for each system with the constant number of particles (*N*), pressure (*P*), and temperature (*T*), i.e., an NPT ensemble. A constant pressure of 1 bar was applied with a coupling constant of 1.0 ps, and water molecules/ions were coupled separately to a bath at 310 K with a coupling constant of 0.1 ps. The leap-frog algorithm^52^ was used for integrating the equation of motion, which was integrated at 2 fs time steps. Production runs were carried out until the root-mean-square deviation (RMSD) value of proteins reached a plateau, indicating equilibration. TOPO I and TOPO II were subjected to 40 and 30 ns production simulations, respectively. The most representative structure was obtained by a cluster tool implemented in GROMACS 5.4.1. Side-chain RMSF calculations were performed by using the rmsf tool in GROMACS 5.4.1.

At the end of molecular docking analyses, the topoisomerase–ligand complexes were subjected to 50 ns classical MD simulations, and binding free energy calculations were performed on the PRODIGY-LIGAND server.^35,36^ Snapshots were taken for every 1 ns of the last 10 ns of the simulations (a total of 10 structures), and they were uploaded to the server. The server calculates the binding free energy by using the following equation:
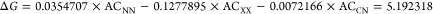
where ACNN, ACCN, and ACXX are the number of atomic contacts (ACs) between nitrogen–nitrogen, carbon–nitrogen, and between all other atoms and polar hydrogens. As described in Vangone et al., the binding free energy is calculated based on the number of atomic contacts (ACs) within the distance threshold of 10.5 Å.^36^ Here, binding energies were predicted for the comparison of various ligands toward the same receptors. These binding free energies were not expected to be the absolute binding free energies for the ligands. The bond lengths between the ligands and their respective binding residues were performed in VMD.^53^ For better observations, interacting heteroatoms (C, O, N, and S) were selected on ligand and interaction residues.

#### Molecular Docking Simulations

Molecular docking simulations were performed by the AutoDock Vina 1.1.2 program^34^ implemented in the YASARA structure software.^33^ Briefly, the most representative protein structures obtained from the MD simulations were placed in a grid box with the dimensions of 64 Å × 64 Å × 64 Å and 71 Å × 71 Å × 71 Å for TOPO I and TOPO II, respectively. This ensured that the whole protein–DNA complex was covered for ligand docking. The spacing was arranged to 1.00 Å, and exhaustiveness was set to 20. Twenty poses were obtained, and then, they were clustered according to their binding mode and binding affinities. The greatest number of poses (approximately 70% of the total) was observed for all the compounds in the DNA binding sites of TOPO I and II. Consequently, the DNA binding sites of TOPO I and II are identified as the primary binding sites for these compounds, with the exception of **SelB** binding to TOPO I. In the docking analysis, the clusters for **SelB** were equally distributed across two distinct sites in TOPO I. Therefore, these two sites are considered potential binding sites for TOPO I.

### Annexin V and PI Assays

PC-3 cells were seeded to a 6-well cell culture plate with a concentration of 5.5 × 10^5^. After cell attachment, cells were treated with determined doses of **SelB-1** (IC_50_ and 2-fold IC_50_) for 48 h. Then, cells were washed with PBS and collected with trypsin. Cells were stained with a Guava Nexin reagent and propidium iodide (PI). Then, samples were analyzed using a flow cytometer and analyzed with CPX software (Beckman Coulter FC500 system, USA).

### Gene Expression Analysis

The total RNA samples from PC-3 cells were isolated by using a total RNA purification plus kit, and total RNA concentrations were measured with a Qubit RNA BR assay kit according to the protocols presented by the manufacturer. cDNA synthesis of these RNA samples was performed by using the high-xapacity cDNA reverse transcription kit. The quantitative gene expression levels of LC-3 (forward: 5′-GAGAAGCAGCTTCCTGTTCTGG-3′, reverse: 5′-GTGTCCGTTCACCAACAGGAAG-3′), Beclin-1 (forward: 5′-TGTCACCATCCAGGAACTCA-3′, reverse: 5′-CTGTTGGCACTTTCTGTGGA-3′), ATG-3 (forward: 5′-TCACAACACAGGTATTACAGG-3′, reverse: 5′-TCACCGCCAGCATCAG-3′), ATG-5 (forward: 5′-GGGAAGCAGAACCATACTATTTG-3′, reverse: 5′-AAATGTACTGTGATGTTCCAAGG-3′), ATG-7 (forward: 5′-AGGAGATTCAACCAGAGACC-3′, reverse: 5′-GCACAAGCCCAAGAGAGG-3′), and ATG-12 (forward: 5′-TCTATGAGTGTTTTGGCAGTG-3′, reverse: 5′-ATCACATCTGTTAAGTCTCTTGC-3′) genes were analyzed using specific TaqMan gene expression assay probes and a TaqMan fast advanced master mix by using an Applied Biosystems 7500 real-time PCR system (Applied Biosystems, Thermo Fisher Scientific). The human-β-2 gene (forward: 5′-TTTCTGGCCTGGAGGCTATC-3′, reverse: 5′-ATGTCTCCATCCCACTTAACT-3′) was selected as a housekeeping gene. The effects of different doses of compounds on gene expression levels were evaluated via the comparative ΔΔCt method.^54^

### GSH Assay and MDA Assay

GSH and MDA assays were performed to evaluate effect of the **SelB-1** on ferroptotic cell death. IC_50_ values of the etoposide on PC-3 cells were determined to be 26.5 μM.^55^ Furthermore, a 25 μM application leads to an ∼20% decrease in the GSH level of PC-3 cells after 24 h of application.^56^ For this reason, ETP was used as a positive control in GSH and MDA assays. The PC-3 cells were seeded to a 6-well cell culture plate with a concentration of 5.5 × 10^5^. After cell attachment, cells were treated with determined doses of **SelB-1** (IC_50_ and 2-fold IC_50_) for 48 h. Total protein samples were isolated from cells by using a RIPA buffer. The concentration of total protein lysates was determined by the BCA assay. Then, 30 μL of a 0.20 M Tris buffer (20 mM EDTA, pH:8.2), 10 μL of a protein sample (10 μg/well) or GSH standards, 20 μL of 0.01 M 5,5′-dithiobis(2-nitrobenzoic acid) (DTNB), and 140 μL of a methanol ACS reagent (≥99.8%) were mixed in 96-well plates. Subsequently, the plate was incubated at 25 °C for 30 min. After incubation, the absorbances (405 nm) were measured using a microplate reader (Tecan Infinite 200 PRO, Switzerland). The relative GSH concentration was calculated according to the standard curve.

For the MDA assay, PC-3 cells were seeded to a 6-well cell culture plate with a concentration of 5.5 × 10^5^. After cell attachment, cells were treated with determined doses of **SelB-1** (IC_50_ and 2-fold IC_50_) for 48 h. Following this treatment, the cells were harvested in a growth medium and centrifuged. The resulting pellet was suspended in a solution containing 20% trichloroacetic acid and subsequently centrifuged again at 15,000*g* for 30 min. The supernatant from the centrifuged cells was collected and combined with a 0.8% thiobarbituric acid solution. This mixture was incubated at 90 °C for 1 h. After incubation, the absorbance at 565 nm was measured utilizing a microplate reader (Tecan Infinite 200 PRO, Switzerland). The MDA concentration was calculated using the below formula:
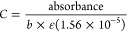


*C* is the MDA concentration (nmol/mg protein). *b* × ε = light path × equivalent value.

### Statistical Analysis

All data were reported as means ± SEM of three independent biological replicates, and differences compared to control groups were analyzed by one-way ANOVA with Dunnett’s post hoc test by using GraphPad Prism 8 software (GraphPad Software, Inc., San Diego, CA) (*p < 0.02, **p < 0.005, and ***p < 0.001).

## Supporting Information Available

Effect of **SelB-1** human colon, breast, and prostate cancer cell lines and a healthy cell line (Figure S1); effect of **SelB-1** on would healing and colony formation capabilities of colon cancer cells (Figure S2); RMSD values of the TOPO I and TOPO II at the end of MD simulations (Figure S3); RMSD values of compound–protein complexes after 50 ns MD simulations (Figures S4 and S5); ligand RMSD values during 50 ns MD simulations (Figures S6 and S7); binding mode and interactions between the compounds and TOPO I after molecular docking simulations (Figure S8); hydrogen bonding and hydrophobic interaction length change of compounds and their respective binding residues at the end of MD simulations (Figure S9); RMSF value of TOPO I at the end of MD simulations (Figure S10); binding mode and interactions between the compounds and TOPO II after molecular docking simulations (Figure S11); hydrogen bonding and hydrophobic interaction length change of compounds and their respective binding residues at the end of MD simulations (Figure S12); RMSF value of TOPO II at the end of MD simulations (Figure S13); effect of **SelB-1** on cell cycle arrest (Figure S14) (DOCX)

## ABBREVIATIONS

ATG-3: autophagy-related 3
ATG-5: autophagy-related 5
ATG-7: autophagy-related 7
ATG-12: autophagy-related 12
BECN1: Beclin-1
BR: broad range
DMEM: Dulbecco’s modified Eagle medium
DMSO: dimethyl sulfoxide
DOX: doxorubicin
ETP: etoposide
FBS: fetal bovine serum
GSH: l-glutamine
HT-29: human colorectal adenocarcinoma cell line
HUVEC: human umbilical vein endothelial cell line
IC_50_: half-maximal inhibitory concentration
LC-3: microtubule-associated protein 1 light chain 3
MCF-7: human breast cancer cell line
MD: molecular dynamics
PC-3: human prostate cancer cell line
PI: propidium iodide
RMSD: root-mean-square deviation
RMSF: root-mean-square fluctuation
SelA: selagibenzophenone A
SelB: selagibenzophenone B
SI: selectivity index
SPC: simple point charge
SRB: sulforhodamine B
TOPO I: topoisomerase I
TOPO II: topoisomerase II
TPT: topotecan
