# Supplementary material for: Selagibenzophenone B and Its Derivatives: SelB-1, a Dual Topoisomerase I/II Inhibitor Identified through In Vitro and In Silico Analyses
Source: ACS Bio Med Chem Au. 2024 Jul 26;4(4):178–89. doi: 10.1021/acsbiomedchemau.4c00027 (PMC11342340; doi:10.1021/acsbiomedchemau.4c00027)
Supplement: Supplementary file 1 — bg4c00027_si_001.docx [file bg4c00027_si_001.docx]

**Supporting Information**

**Selagibenzophenone B and its derivatives: SelB-1, a dual topoisomerase I/II inhibitor identified through *in vitro* and *in silico* analyses,**

Serhat Dönmez^1^, Ringaile Lapinskaite^2,3^, Hazal Nazlican Atalay^1^, Esra Tokay^4^, Feray Kockar^4^, Lukas Rycek^2^*, Mehmet Özbil^5^, Tugba Boyunegmez Tumer^6^*

^1^ Graduate Program of Molecular Biology and Genetics, School of Graduate Studies, Canakkale Onsekiz Mart University, Canakkale 17020, Turkey

^2^ Department of Organic Chemistry, Center for Physical Sciences and Technology (FTMC), Akademijos g. 7, Vilnius LT-08412, Lithuania

^3^ Department of Organic Chemistry, Faculty of Science, Charles University, Praha 128 00, Czech Republic

^4^ Department of Molecular Biology and Genetics, Faculty of Sciences and Arts, Balikesir University, Balikesir 10145, Turkey

^5^ Institute of Biotechnology, Gebze Technical University, Kocaeli 41400, Turkey

^6^ Department of Molecular Biology and Genetics, Faculty of Science, Canakkale Onsekiz Mart University, Canakkale 17020, Turkey.

Corresponding Author:

Tugba Boyunegmez Tumer: [tumertb@comu.edu.tr](mailto:tumertb@comu.edu.tr), [tumertb@gmail.com](mailto:tumertb@gmail.com)

Lucas Rycek: [rycekl@natur.cuni.cz](mailto:rycekl@natur.cuni.cz)

**Table of Contents**

[**Figure S1.** Effect of **SelB** and **SelB-1** on HT29 (human colon cancer, A), PC-3 (human prostate cancer, B), MCF-7 (human breast cancer, C) and HUVEC (human endothelial cell lines, D). S3](#_Toc169179125)

[**Figure S2.** **SelB-1** inhibited wound recovery (A) and colony formation (B) of HT-29 cells. S4](#_Toc169179126)

[**Figure S3.** The RMSD value of the TOPO I (A) and TOPO II (B) at the end of MD simulations. S5](#_Toc169179127)

[**Figure S4.** The RMSD value of **SelB** compounds (A), **SelA** compounds and **TPT** (B) bounded TOPO I after 50 ns MD simulations. S5](#_Toc169179128)

[**Figure S5.** The RMSD value of **SelB-1**, **SelA-4**, **SelA-3**, and **ETP** bounded TOPO II after 50 ns MD simulations. S6](#_Toc169179129)

[**Figure S6.** The ligand RMSD value of two poses of **SelB** (A), **SelB-1** and **TPT** (B), and SelA compounds (C) bounded TOPO I after 50 ns MD simulations. S6](#_Toc169179130)

[**Figure S7.** The ligand RMSD value of **SelB-1** and **ETP** (A), and **SelA** compounds (B) bounded TOPO II after 50 ns MD simulations. S7](#_Toc169179131)

[**Figure S8.** Binding mode (A) and interactions (B) between **SelB**, **SelB-1**, **SelA-4**, **SelA-3**, **TPT** and TOPO I at molecular docking. S8](#_Toc169179132)

[**Figure S9.** The Hydrogen bonding and hydrophobic interaction length change of **SelB-1st** (A), **SelB-2nd** (B), **SelB-1** (C), **SelA-3** (D), **SelA-4** (E), and **TPT** (F) and their respective binding residues on TOPO I during 50 ns MD simulations. S9](#_Toc169179133)

[**Figure S10.** The RMSF values of **SelB-1st** (A), **SelB-2nd** (B), **SelB-1** (C), **SelA-3** (D), **SelA-4** (E), and **TPT** (F) bounded and unbounded TOPO I. S10](#_Toc169179134)

[**Figure S11.** Binding mode (A) and interactions (B) between **SelA-3**, **SelA-4**, **SelB-1**, **ETP** and TOPO II at molecular docking. S11](#_Toc169179135)

[**Figure S12.** The Hydrogen bonding and hydrophobic interaction length change of **SelB-1** (A), **SelA-3** (B), **SelA-4** (C), and **ETP** (D) and their respective binding residues on TOPO II during 50 ns MD simulations. S12](#_Toc169179136)

[**Figure S13.** The RMSF values of **SelB** (A), **SelA-3** (B), **SelA-4** (C), and **ETP** (D) bounded and unbounded TOPO II. S13](#_Toc169179137)

**Figure S14.** **SelB-1** did not induced cell cycle arrest on PC-3 cells after 48 h. S14


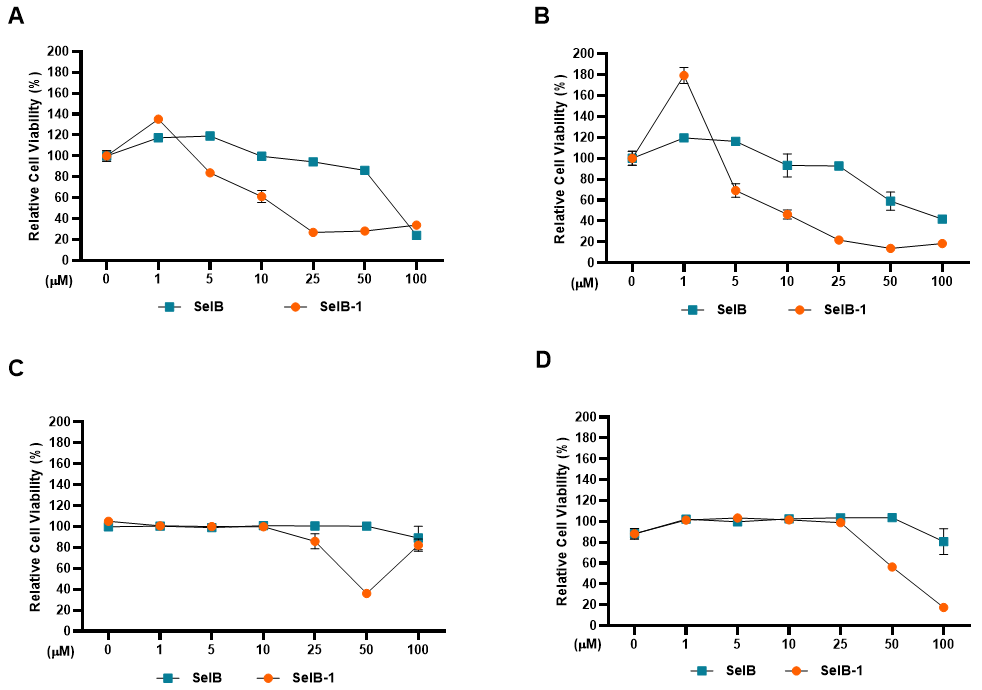


Figure S1. Effect of SelB and SelB-1 on HT29 (human colon cancer, A), PC-3 (human prostate cancer, B), MCF-7 (human breast cancer, C) and HUVEC (human endothelial cell lines, D). Each data point is represented as the mean ± SEM obtained from three independent experiments. SEM: Standard Error of Mean.


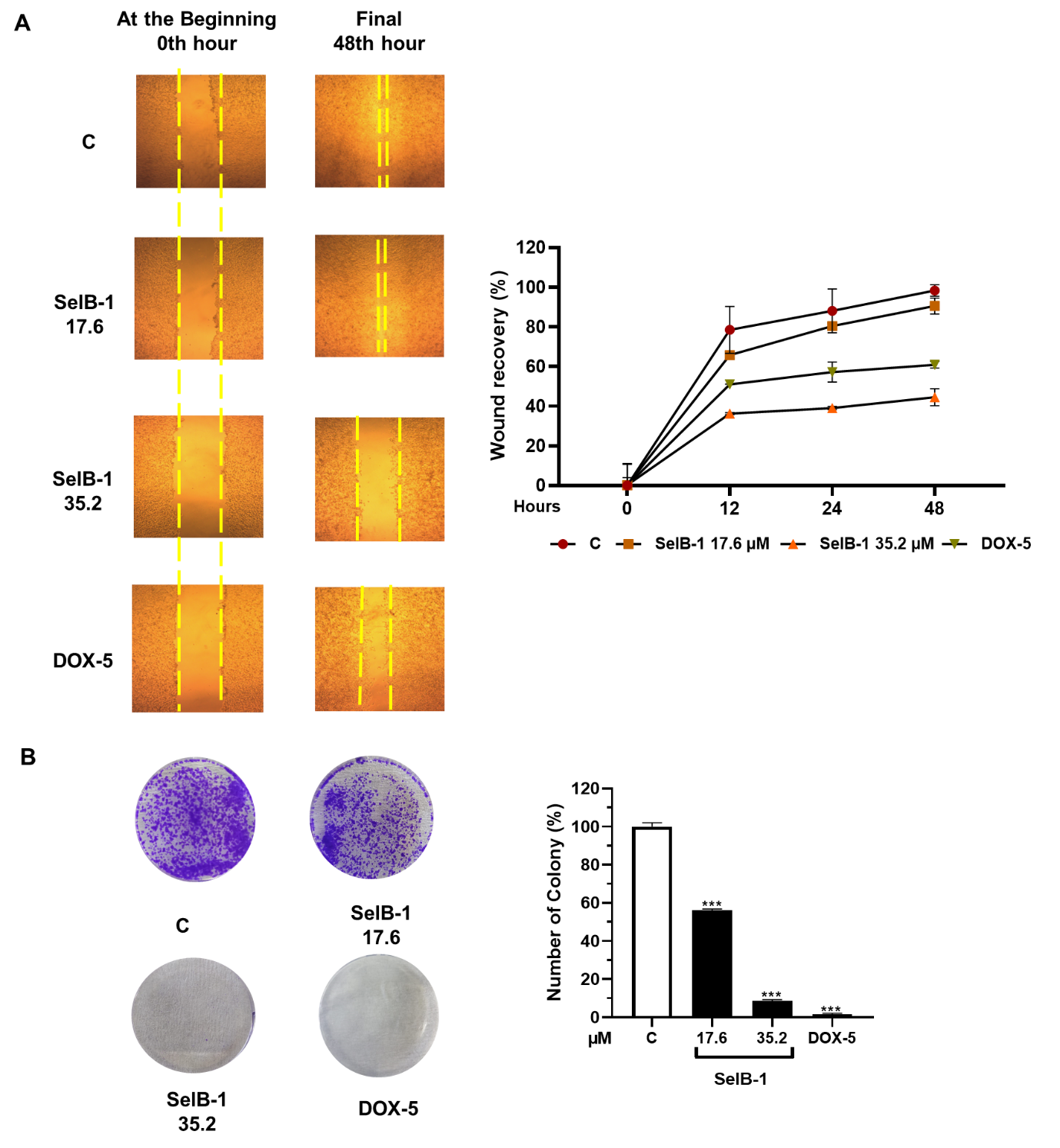


Figure S2. SelB-1 inhibited wound recovery (A) and colony formation (B) of HT-29 cells. C: Control (only DMSO). DOX-5: Doxorubicin 5 µM. Each data point is represented as the mean ± SEM obtained from three independent experiments. SEM: Standard Error of Mean. *p < 0.02, **p < 0.005, ***p < 0.001.


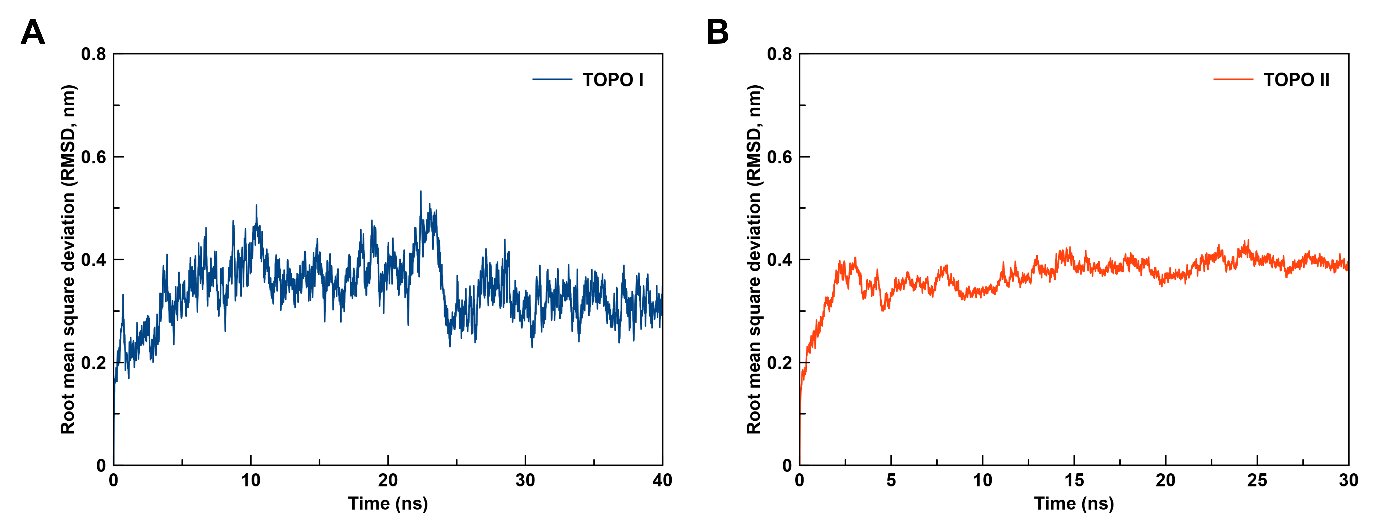


Figure S3. The RMSD value of the TOPO I (A) and TOPO II (B) at the end of MD simulations.


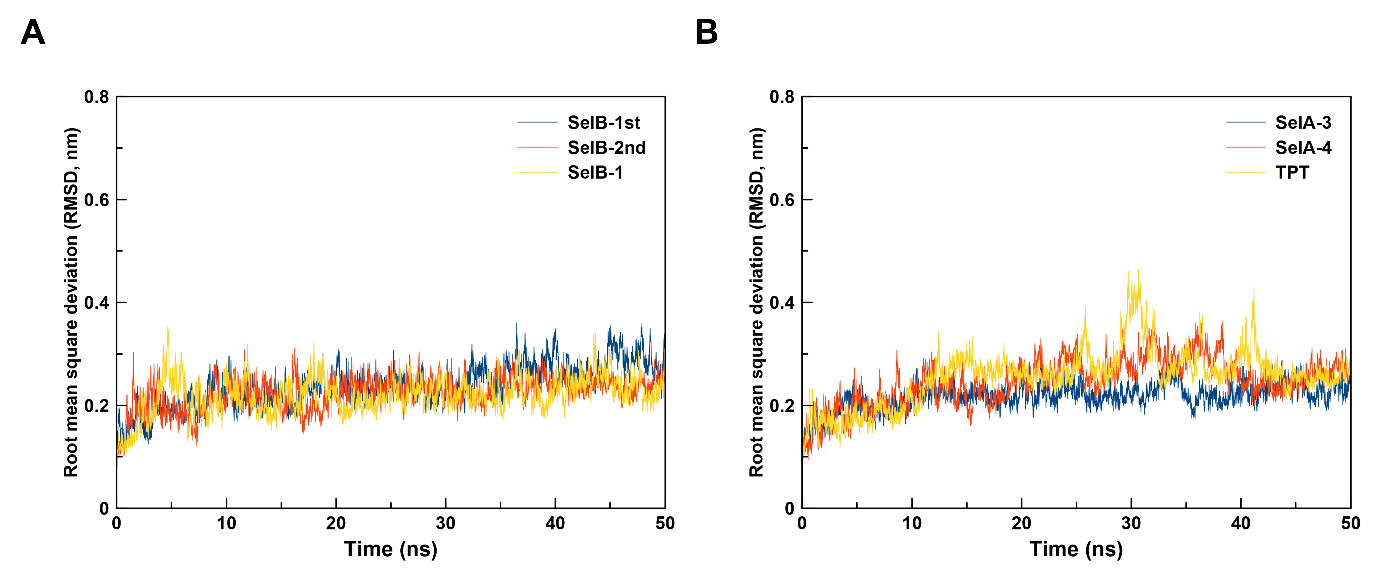


Figure S4. The RMSD value of SelB compounds (A), SelA compounds and TPT (B) bounded TOPO I after 50 ns MD simulations.


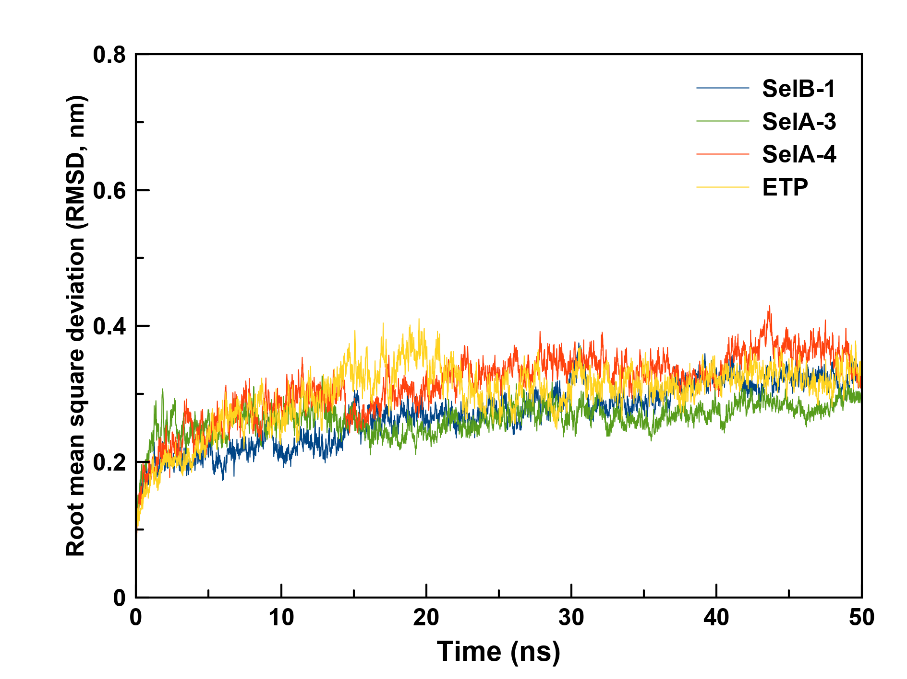


Figure S5. The RMSD value of SelB-1, SelA-4, SelA-3, and ETP bounded TOPO II after 50 ns MD simulations.


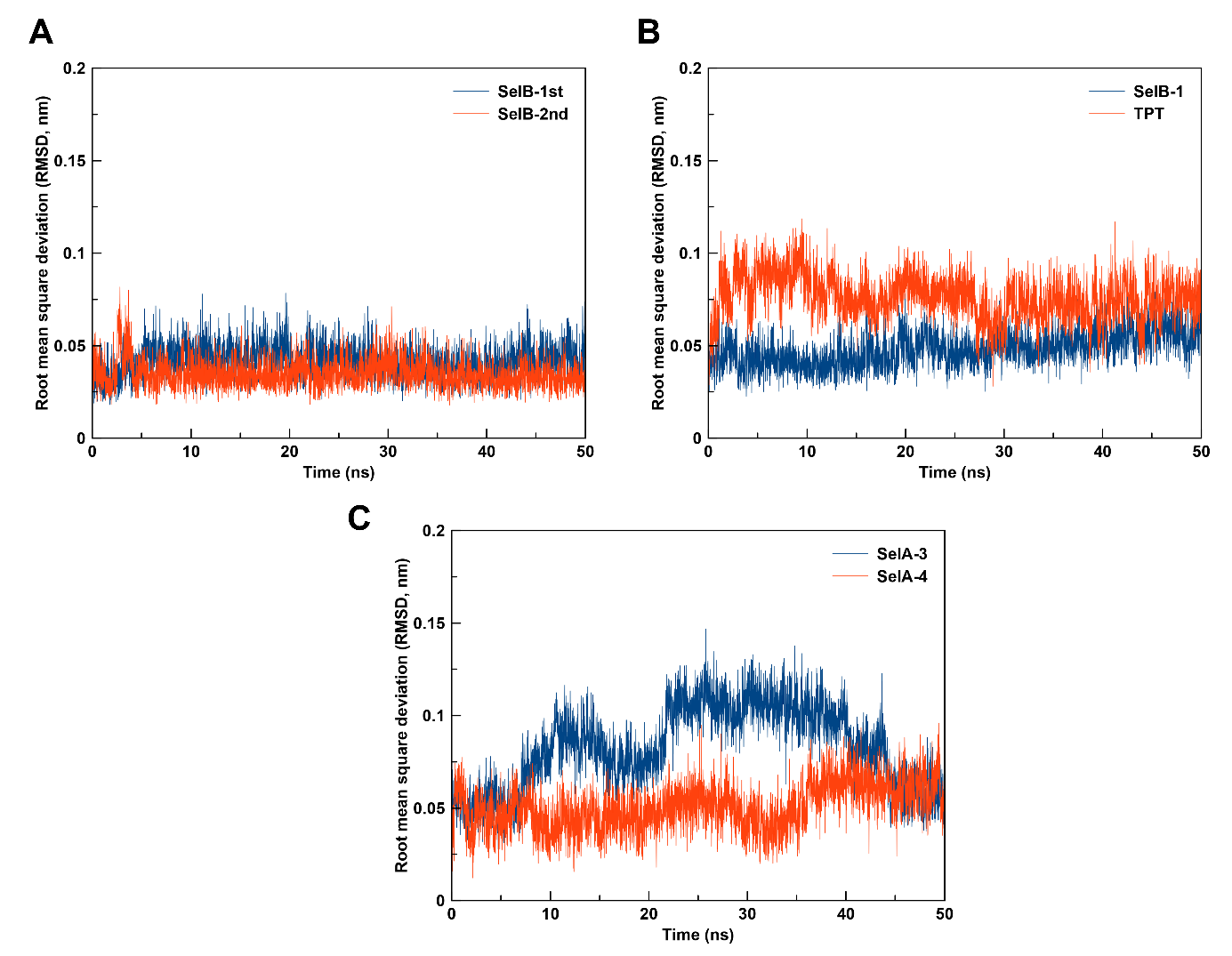


Figure S6. The ligand RMSD value of two poses of SelB (A), SelB-1 and TPT (B), and SelA compounds (C) bounded TOPO I after 50 ns MD simulations.


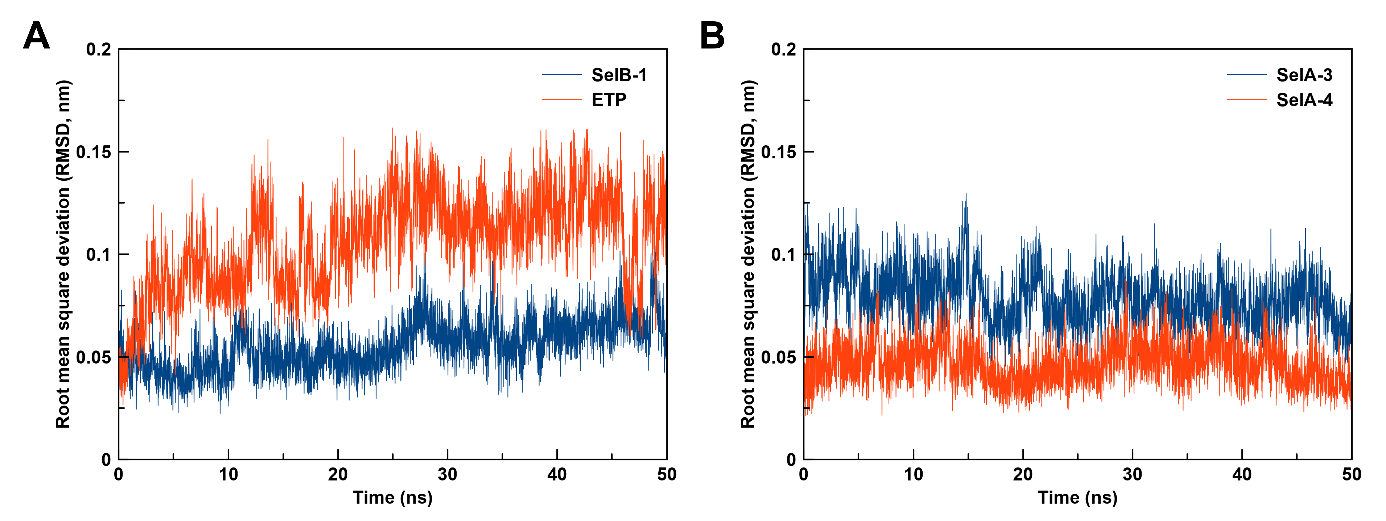


Figure S7. The ligand RMSD value of SelB-1 and ETP (A), and SelA compounds (B) bounded TOPO II after 50 ns MD simulations.


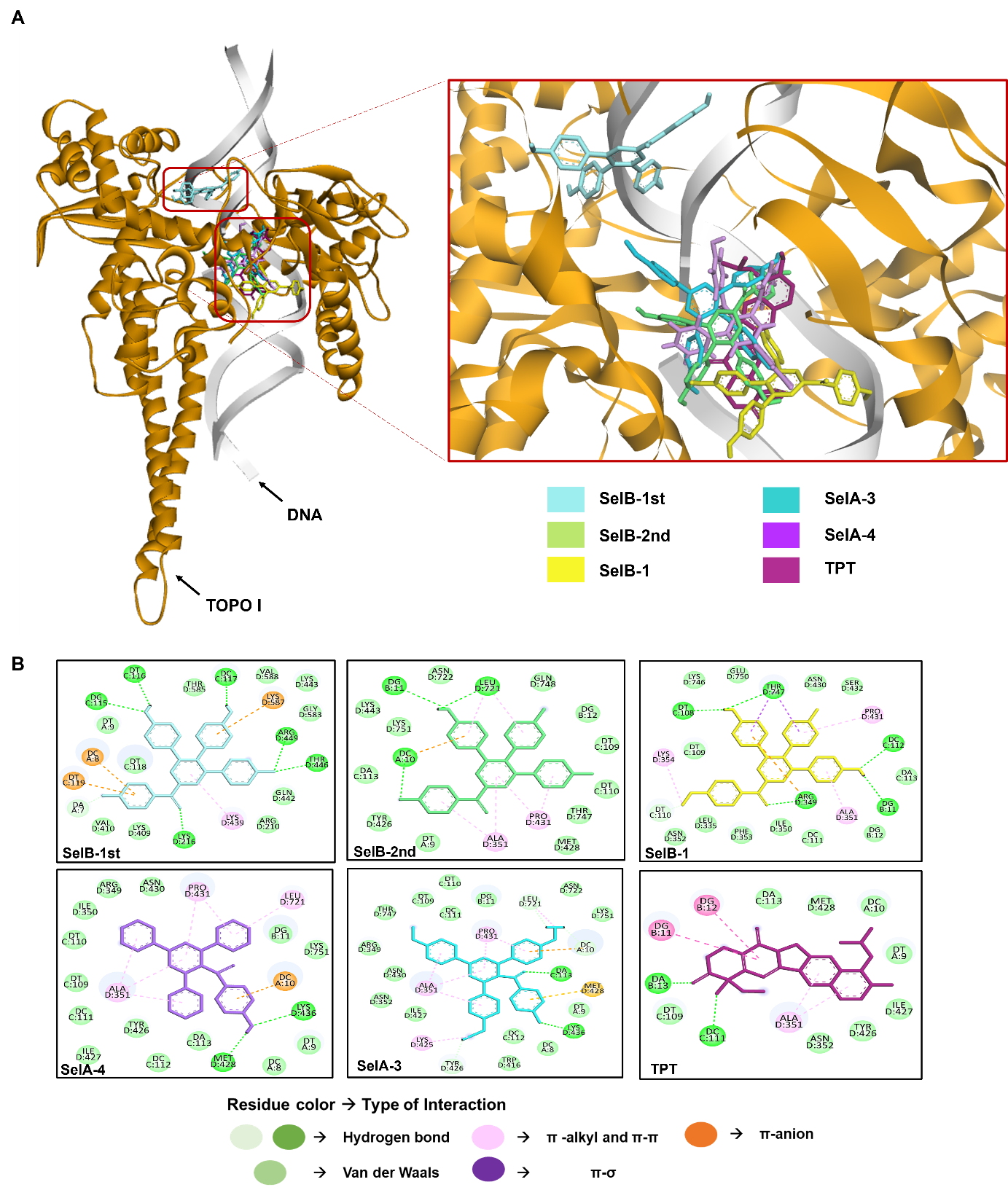


Figure S8. Binding mode (A) and interactions (B) between SelB, SelB-1, SelA-4, SelA-3, TPT and TOPO I at molecular docking.


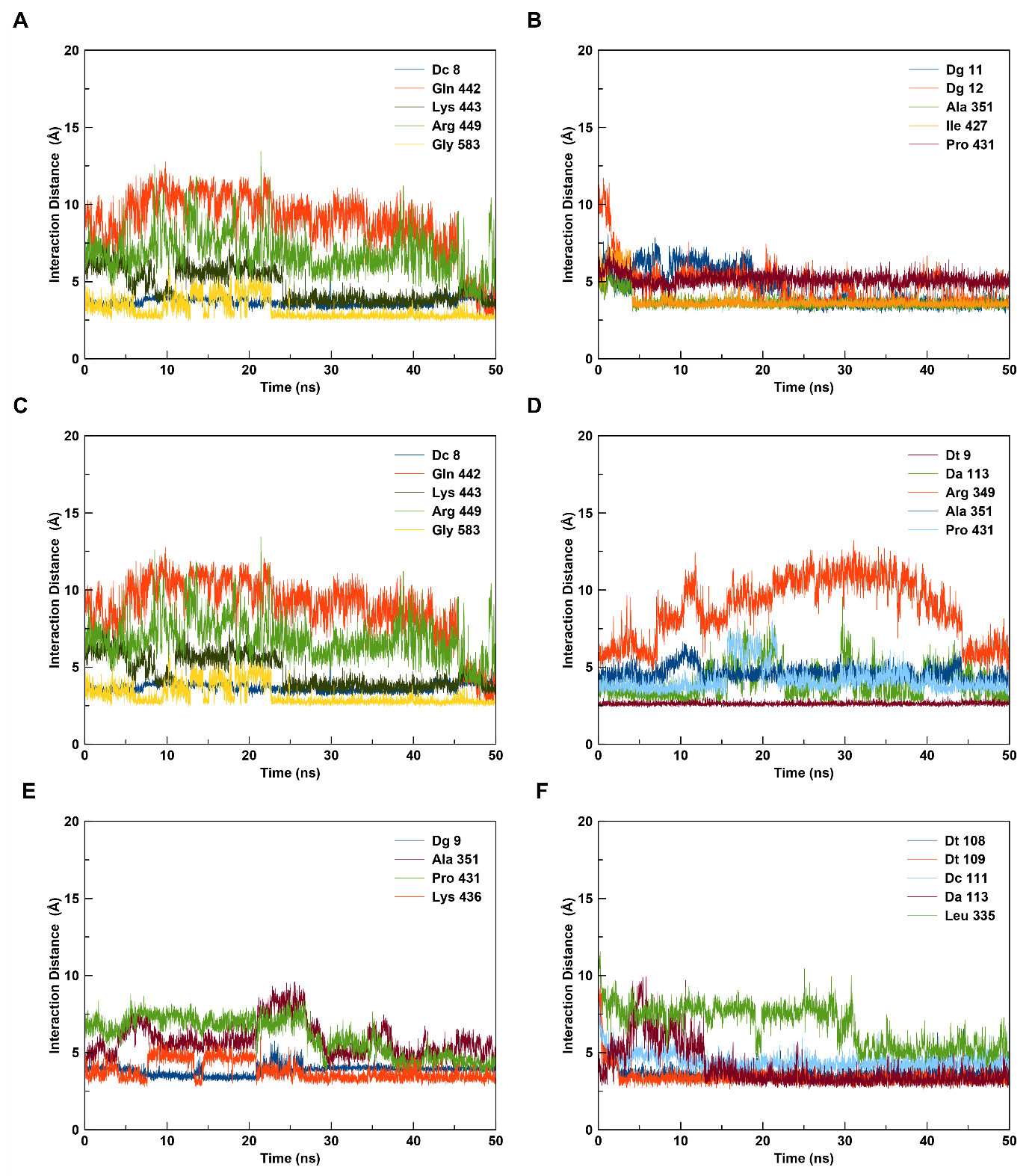


Figure S9. The Hydrogen bonding and hydrophobic interaction length change of SelB-1st (A), SelB-2nd (B), SelB-1 (C), SelA-3 (D), SelA-4 (E), and TPT (F) and their respective binding residues on TOPO I during 50 ns MD simulations.


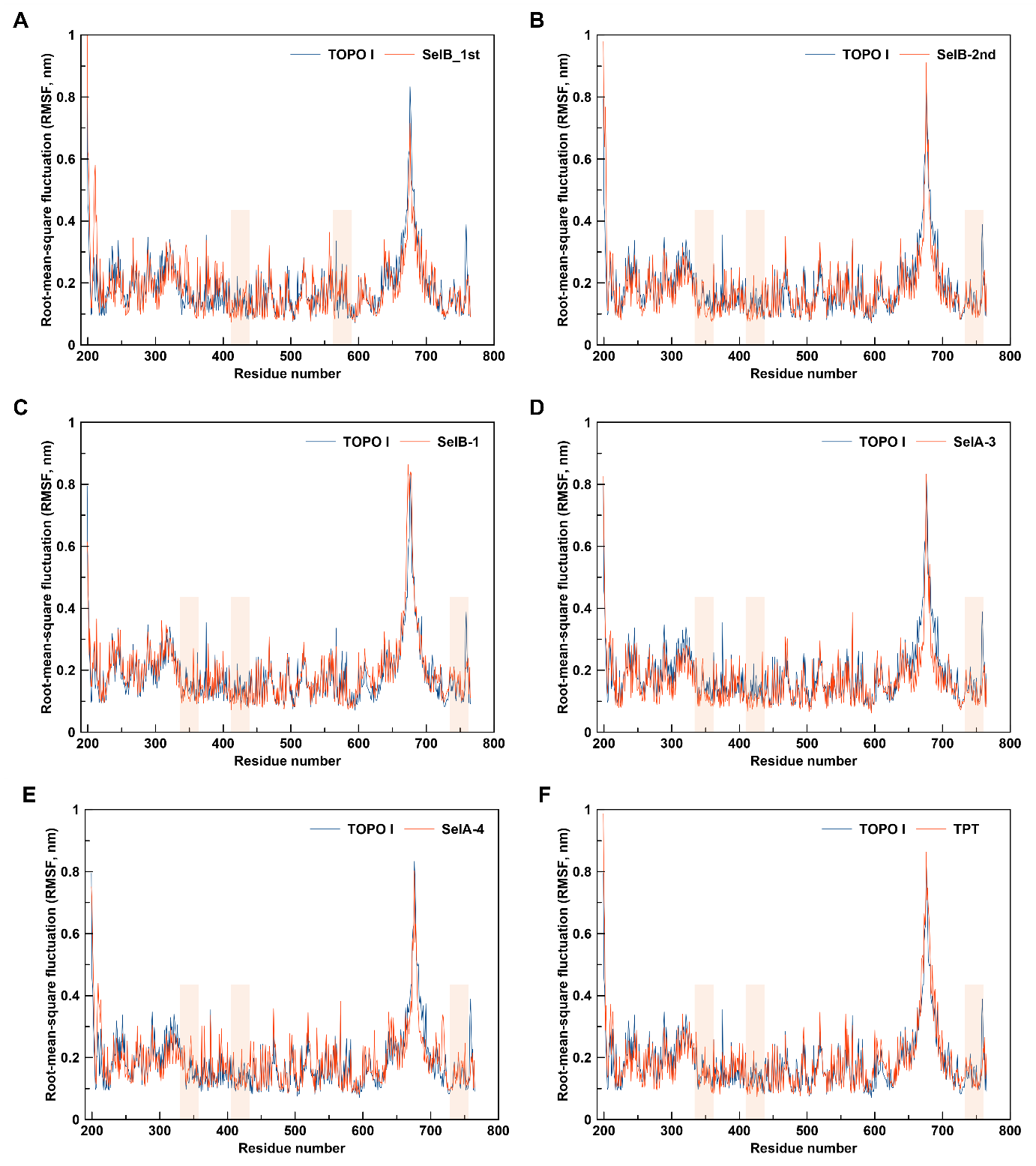


Figure S10. The RMSF values of SelB-1st (A), SelB-2nd (B), SelB-1 (C), SelA-3 (D), SelA-4 (E), and TPT (F) bounded and unbounded TOPO I. The orange boxes shows the binding sites of the compounds on TOPO I.

**
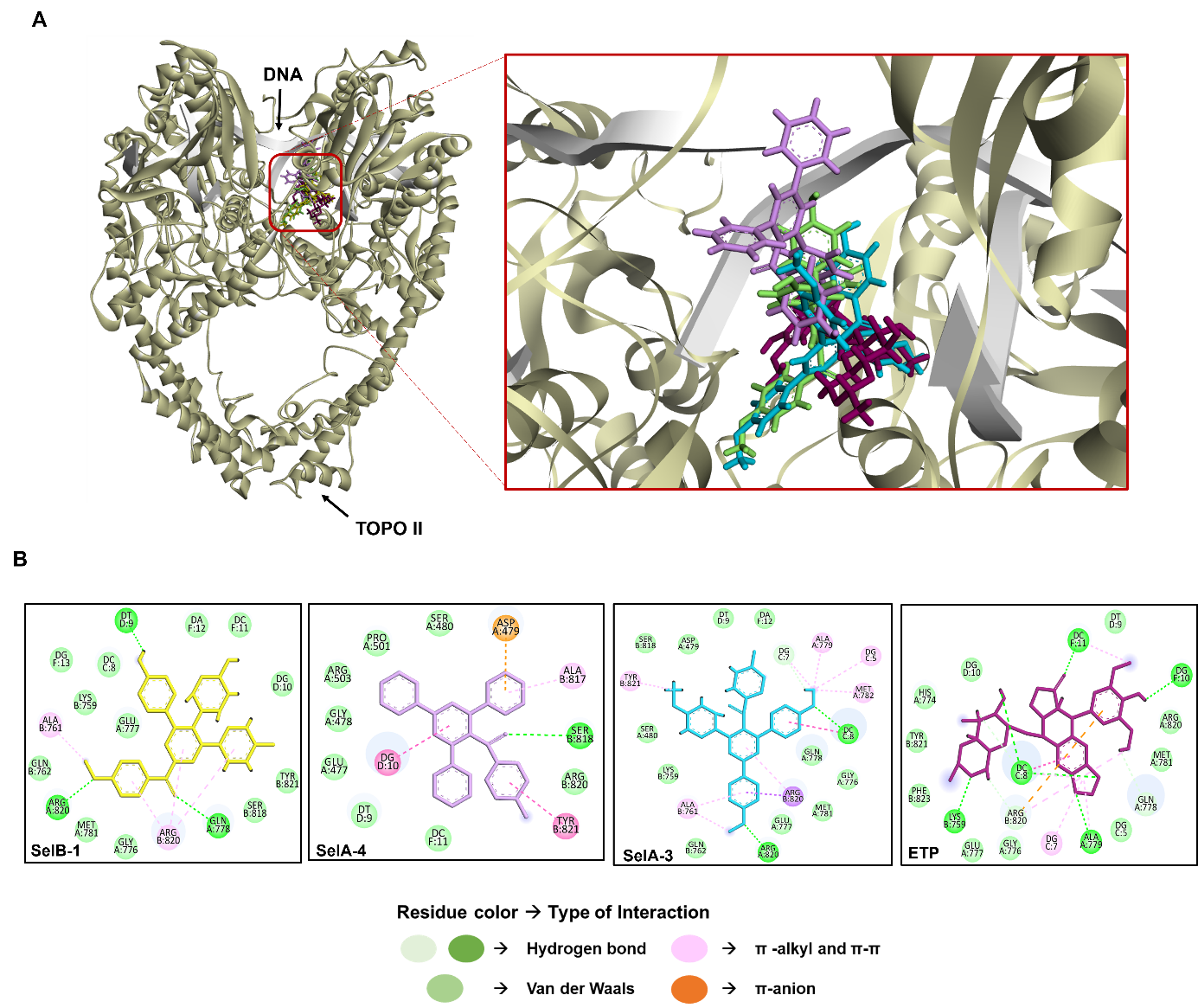
**

Figure S11. Binding mode (A) and interactions (B) between SelA-3, SelA-4, SelB-1, ETP and TOPO II at molecular docking.


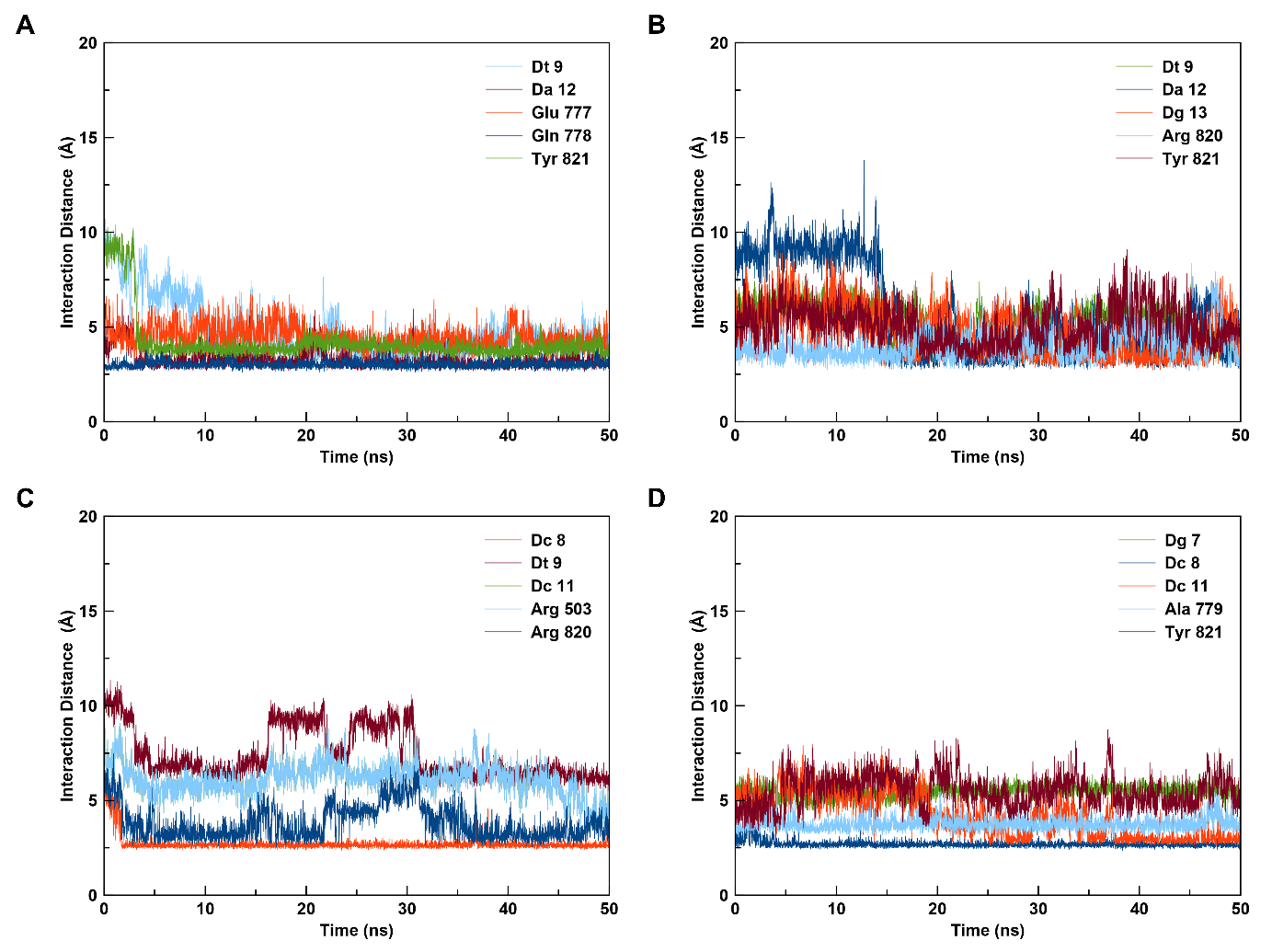


Figure S12. The Hydrogen bonding and hydrophobic interaction length change of SelB-1 (A), SelA-3 (B), SelA-4 (C), and ETP (D) and their respective binding residues on TOPO II during 50 ns MD simulations.


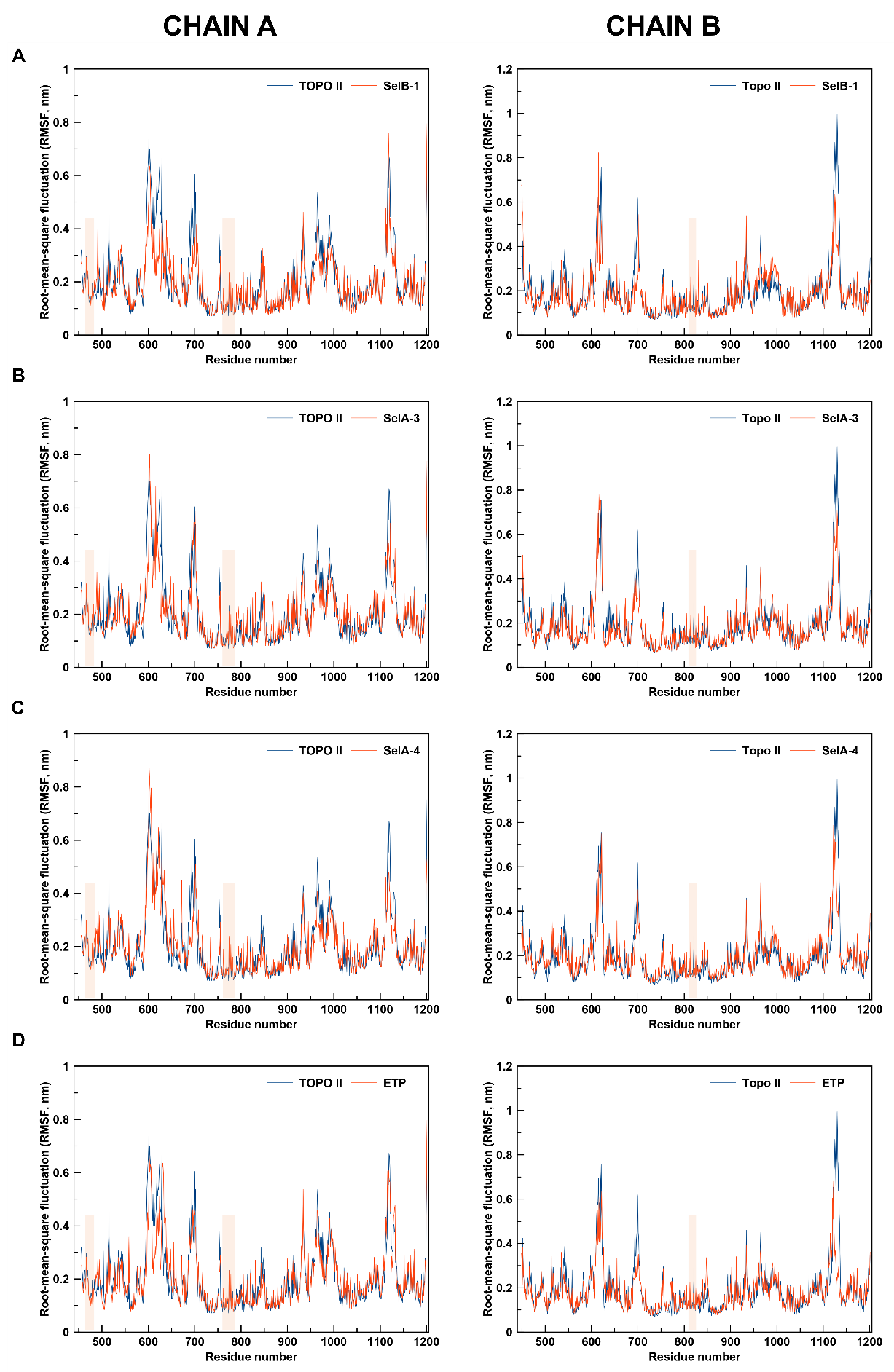


Figure S13. The RMSF values of SelB (A), SelA-3 (B), SelA-4 (C), and ETP (D) bounded and unbounded TOPO II. The orange boxes shows the binding sites of the compounds on TOPO II.


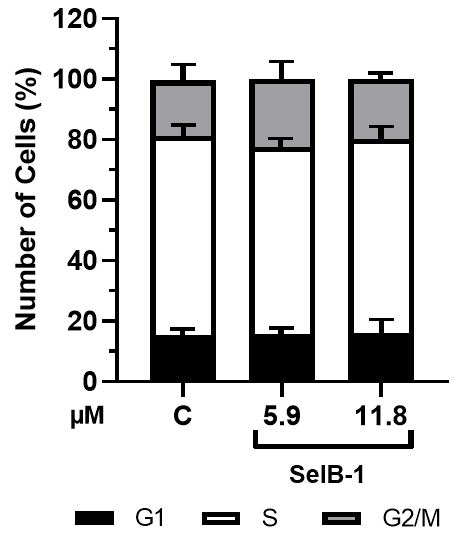


|  |  | **SelB-1** | |
| --- | --- | --- | --- |
|  | **C** | **5.9 µM** | **11.8 µM** |
| **G1 (%)** | 15.3 | 15.5 | 16.2 |
| **S (%)** | 65.9 | 62 | 64.1 |
| **G2 (%)** | 18.3 | 22.5 | 19.7 |

Figure S14. SelB-1 did not induced cell cycle arrest on PC-3 cells after 48 h. C: Control (0.1 % DMSO).
